# A novel lncRNA ARST represses glioma progression by inhibiting ALDOA-mediated actin cytoskeleton integrity

**DOI:** 10.1186/s13046-021-01977-9

**Published:** 2021-06-07

**Authors:** Jun Sun, Dong He, Yibing Fu, Rui Zhang, Hua Guo, Zhaojuan Wang, Yanan Wang, Taihong Gao, Yanbang Wei, Yuji Guo, Qi Pang, Qian Liu

**Affiliations:** 1 Department of Neurosurgery, Shandong Provincial Hospital, Cheeloo College of Medicine, Shandong University, Jinan, 250012 Shandong People’s Republic of China; 2 Department of Histology and Embryology, School of Basic Medical Science, Cheeloo College of Medicine, Shandong University, Jinan, 250012 Shandong People’s Republic of China; 3 Department of Obstetrics and Gynecology, Shandong Provincial Hospital, Cheeloo College of Medicine, Shandong University, Jinan, 250012 Shandong People’s Republic of China; 4 Department of Physiology, Shandong Medical College, Jinan, 250012 Shandong People’s Republic of China; 5 Department of Pathology, Tai-an Municipal Hospital, Jinan, 250012 Shandong People’s Republic of China

**Keywords:** LncRNA, Glioma, ARST, ALDOA, Actin, Cytoskeleton

## Abstract

**Background:**

Glioma is one of the most aggressive malignant brain tumors that is characterized with inevitably infiltrative growth and poor prognosis. ARST is a novel lncRNA whose expression level is significantly decreased in the patients with glioblastoma multiforme. However, the exact mechanisms of ARST in gliomagenesis are largely unknown.

**Methods:**

The expressions of ARST in the glioma samples and cell lines were analyzed by qRT-PCR. FISH was utilized to detect the distribution of ARST in the glioma cells. CCK-8, EdU and flow cytometry were used to examine cellular viability, proliferation and apoptosis. Transwell and wound-healing assays were performed to determine the migratory and invasive abilities of the cells. Intracranial tumorigenesis models were established to explore the roles of ARST in vivo. RNA pulldown assay was used to examine proteins that bound to ARST. The activities of key enzymes in the glycolysis and production of lactate acid were measured by colorimetry. In addition, RIP, Co-IP, western blot and immunofluorescence were used to investigate the interaction and regulation between ARST, F-actin, ALDOA and cofilin.

**Results:**

In this study, we reported that ARST was downregulated in the gliomas. Overexpression of ARST in the glioma cells significantly suppressed various cellular vital abilities such as cell growth, proliferation, migration and invasion. The tumorigenic capacity of these cells in vivo was reduced as well. We further demonstrated that the tumor suppressive effects of ARST could be mediated by a direct binding to a glycolytic enzyme aldolase A (ALDOA), which together with cofilin, keeping the polymerization and depolymerization of actin filaments in an orderly dynamic equilibrium. Upregulation of ARST interrupted the interaction between ALDOA and actin cytoskeleton, which led to a rapid cofilin-dependent loss of F-actin stress fibers.

**Conclusions:**

Taken together, it is concluded that ARST performs its function via a non-metabolic pathway associated with ALDOA, which otherwise modifies the morphology and invasive properties of the glioma cells. This has added new perspective to its role in tumorigenesis, thus providing potential target for glioma diagnosis, therapy, and prognosis.

**Supplementary Information:**

The online version contains supplementary material available at 10.1186/s13046-021-01977-9.

## Background

Gliomas are the most common and aggressive malignant brain tumors, which account for 30% of all central nervous system tumors and 80% of all primary malignant brain tumors [1]. Gliomas are characterized with inevitably infiltrative growth, making them elusive targets for effective surgical management and leading to postoperative recurrence. According to the pathology, Gliomas are classified into four grades: I, II, III and IV [2]. Grade II and III, are referred to the Low Grade Gliomas (LGGs), while Glioblastoma Multiforme (GBM) is the most severe glioma type that belongs to Grade IV. Despite advances in neuro-oncology to optimize treatment options, the prognosis of patients with gliomas still remains unsatisfactory. Therefore, it is critical to understand the pathogenesis of gliomas and explore new effective therapeutic targets for better clinical inventions.

Recently, the roles of non-coding RNAs (ncRNAs) in tumors have attracted increasing attentions [3], among which the long non-coding RNAs (lncRNAs) are a diverse and poorly conserved category of RNAs [4]. With the length longer than 200 nucleotides, lncRNAs exert crucial roles on the transcriptional and post-transcriptional regulation of gene expression [5]. Nowadays, accumulating evidences have indicated the close involvement of lncRNAs in cancer pathogenesis, including gliomas. For instance, lncRNA MIR22HG is a critical inducer of the Wnt/β-catenin signaling pathway, which provides a potential therapeutic strategy for glioma patients by targeting the lncRNA MIR22HG [6]. What’s more, the lncRNA HOTAIR contained in the serum-derived exosomes can also be used as a novel prognostic and diagnostic biomarker for GBM [7]. Until now, more and more glioma-associated lncRNAs have been discovered, although their functions and regulatory mechanisms are still elusive.

Fructose-bisphosphate aldolase A (ALDOA) is one of the key enzymes in glycolysis. Since the most well-known and prevalent metabolic change associated with cancer cells is enhanced aerobic glycolysis, ALDOA is found to be highly expressed in many types of cancers acting as the oncogene, including renal clear cell, hepatocellular and lung squamous cell carcinomas [8–10]. What’s more, the increasing level in ALDOA expression is positively correlated with the degree of malignancy and inversely correlated with the prognosis. Most remarkably, the latest research proposed that several glycolytic enzymes exerted moonlighting roles that are independent of glycolysis and they acted as regulators of a variety of cellular processes. ALDOA, for instance, plays an indispensable role in the polymerization of actin cytoskeleton [11].

Actin filament is one of the key elements of cytoskeleton structure, and its integrity is crucial for various cellular processes such as proliferation, migration, invasion and apoptosis [12]. In normal circumstances, a dynamic equilibrium state is maintained between depolymerization of mature actin filaments and polymerization of new fibers, the cycle of which contributes to the migration and invasion of the cells [13, 14]. Recent studies have clarified that ALDOA could promote F-actin polymerization by interacting with G-actin. Blocking the interaction between ALDOA and F-actin reversed the polymerization to G-actin [11, 15]. On the other hand, cofilin, an actin depolymerization protein (ADP), plays a pivotal role in the depolymerization of actin cytoskeleton [11]. The subsequent release of G-actin monomers would be used to promote the assembly of new F-actin fibers. However, the detailed molecular regulatory mechanisms of this process still need further investigations.

In this study, we reported a novel lncRNA, ALDOA related specific transcript (ARST), which was downregulated in the gliomas. The overexpression of ARST in glioma cells interrupted the interaction between ALDOA and F-actin cytoskeleton, which led to the rapid cofilin-dependent loss of F-actin stress fibers. In addition, various cellular vital abilities such as cell growth, proliferation, migration and invasion were inhibited. This research demonstrated for the first time that ARST acts as a tumor suppressor in GBM by a non-metabolic pathway associated with ALDOA, which may be a potential therapeutic target for glioma diagnoses, therapies, and prognoses.

## Methods

### High throughput sequencing analysis and data arrangement

Using high throughput sequencing technique, we defined the clusters of differentially expressing lncRNAs in the glioma specimens and paracancerous tissues. The suffix E-Signal represented the paracancerous tissues, and the suffix T-Signal represented the tumor tissues. Clusters in green indicated the downregulated lncRNAs and clusters in red indicated the opposite.

### Clinical samples and cell lines

A total of 144 specimens were collected from tumorectomy of gliomas in the Department of Neurosurgery, Shandong Provincial Hospital Affiliated to Shandong University, Jinan. The adjacent brain tissue was defined as 1 cm away from the lesions. The criteria for the inclusion/exclusion of patient are: (1) age 0–70; (2) only received surgeries, without preoperative chemotherapies or radiation therapies; (3) without other types of cancers, autoimmune diseases, infectious diseases, etc. All specimens were obtained under sterile conditions during surgeries, snap frozen in liquid nitrogen, and stored at − 80 °C. The corresponding clinical data were also obtained. This study was approved by the Human Ethics Committee of Hospital. The individuals were informed about the study and gave consent prior to the specimen collection.

We used human glioma cell lines U87MG and U251, normal human astrocytes (NHA) and mouse glioma cell line GL261 as our experimental cell lines, provided from Shanghai Institutes for Biological Sciences Cell Resource Center.

### Cell culture and transfection

The cell lines were cultured in DMEM medium with high glucose and sodium pyruvate, supplemented with 10% fetal bovine serum, 100 units/mL penicillin and 100 μg/mL streptomycin. The culture condition was 37 °C with 5% CO_2_. Transfections of SMART silencer RNA (RiboBio, Guangzhou, China) and the plasmids for overexpression were performed via Lipofectamine 2000 (Invitrogen, CA, USA). Plasmid extraction was conducted via OMEGA Endo-free Plasmid Mini Kit II (OMEGA bio-tek, Guangzhou, China), according to the manufacturer’s instructions. The glycerol bacteria containing ALDOA mutants was produced by Shanghai BioSune Biotechnology Limited Company. Detailed transfection procedures were referred to the manufacturers’ introduction.

### RNA fluorescence in situ hybridization (FISH) assay

In order to detect subcellular localization of lncRNA ARST in the glioma cells, we utilized Fluorescence in Situ Hybridization Kit (Cat.10910) (RiboBio, Guangzhou, China) in the study. Procedures were described previously [16]. The probes were designed and synthesized by RiboBio, Guangzhou, China. The hybridization step was performed in 42 °C overnight with enough humidity.

### RNA isolation and quantitative reverse transcription PCR (qRT-PCR) assay

Experimental related primers were designed and produced by Takara, Japan. Cellular total RNA was isolated and concentrated by TransZol Up (Transgen, Beijing, China). The concentration and purity of RNA were determined by measuring the absorbance at 260 nm and the absorbance ratio of 260/280 nm in a NanoDrop Spectrophotometer (Thermo Scientific, Wilmington, DE, USA). gDNA was removed and cDNA was reversed transcribed by TransScript One-Step gDNA Removal and cDNA synthesis SuperMix (Transgen, Beijing, China). A CFX Connect Real-time PCR System (Bio-rad, CA, USA) and a TransStart Tip Green qPCR SuperMix Kit (Transgen, Beijing, China) were used for real-time PCR. The sequences of the primers were listed below.

**Table:** 

**Gene**	**Forward**	**Reverse**
ARST	5′-TCAGCGCATAGCTCAAGTCT-3’	5′-GGTAGGCTCTTCTCAGGCAC-3′
ALDOA	5′-ATGCCCTACCAATATCCAGCA-3’	5′-GCTCCCAGTGGACTCATCTG-3’
RhoA	5′-GCCGGTGAAACCTGAAGAAG-3’	5′-GCAGCTCTCGTAGCCATTTC-3’
ROCK1	5′-GGAAGTGAGGTTAGGGCGAA-3’	5′-ACAGTGTCTCGGAGCGTTTC-3’
LIMK1	5′-ATGGCCTACCTCCACTCCAT-3’	5′-TTCTTGCGGTCTGGCTTCTT-3’
cofilin	5′-CTGCCGCTATGCCCTCTATG-3’	5′-TCCTTGACCTCCTCGTAGCA-3’

### Cell proliferation assay

Cell viability was determined using the Cell Counting Kit-8 (CCK-8) (Dojindo Laboratories, Shanghai, China). In details, the transfected U87MG and U251 cells were seeded for 0, 24, 48, and 72 h at 2 × 10^3^ cells/well in 96-well plates. Subsequently, 10 μL CCK-8 solution was added to each well and the cells were incubated for 2 h. Absorbance at 450 nm was measured on the iMark Microplate Reader (Bio-rad, CA, USA). The experiment was repeated thrice. Detail procedures were described previously [17].

EdU assay was performed by Cell-Light™ EdU Apollo567 In Vitro Imaging Kit (RiboBio, Guangzhou, China) at 24 h after the transfected 1 × 10^4^ cells were plated into 96-well plates. After EdU labeling, the percentage of EdU-positive cells were visualized under a fluorescence microscope (Olympus, Tokyo, Japan) as described previously [17].

### Wound-healing and transwell assays

To assess the migrative and invasive abilities of glioma cells in vitro, wound-healing and transwell assays were performed. For the wound-healing assay, glioma cells were planted on 6-well cell culture plates (Corning, NY, USA). Scratching step was vertically performed on the center of each well, using 200 μL pipettes. The cells were cultured for 12 h in high glucose DMEM medium without fetal bovine serum (FBS). The pictures were taken on the microscope at 200×. The gap distance was measured by the plotting scale of the software. The proportion of changes were calculated and analyzed in statistics. For transwell assay, cells were cultured in the transwell chambers with 8 μm pores (Corning star, Lowell, MA, USA) in the 24-well plates. For the migration assay, 1000 transfected cells were suspended in 100 μL serum-free medium and added to the upper transwell chamber. After incubation for 6 h in a humidified atmosphere containing 5% CO2 at 37 °C, the migrated cells that had stuck to the lower surface of the membrane were fixed in 4% paraformaldehyde and stained with crystal violet for 45 min. The number of migrated cells was counted in five randomly selected fields at 200× magnification using a microscope. For the invasion assay, the transwell chambers were coated with Matrigel (BD Bioscience, NJ, USA), and same procedures as those for the migration assay were followed [17].

### Flow cytometry assay

Annexin V-FITC Apoptosis Detection Kit (Beyotime Biosciences, Shanghai, China) was applied to detect level of cell apoptosis. The U87MG cells were seeded in a 6-well plate at 2.0 × 10^5^/well for 48 h. Then the cells were digested by 0.08% trypsin and washed with ice-cold PBS for three times. After that, cells were marked with Annexin V-FITC and PI for an incubation of 15 min in darkness. Cytoflex S (Beckman coulter, CA, USA) was utilized to check the staining ratio of FIFC/PI and calculate the level of apoptosis.

### Western blot

Total proteins were extracted using RIPA lysis buffer (1:1000) (Transgen, Beijing, China) and protease/phosphates inhibitors (Apexbio, TX, USA). Concentration of proteins was measured by BCA Protein Quantification Kit (Vazyme, Jiangsu, China). Proteins were separated by 10% or 12.5% SDS-PAGE gel electrophoresis, transferred to PVDF membranes and probed with primary antibodies. The membranes were subsequently probed with horseradish peroxidase-conjugated secondary antibodies. Then, an enhanced chemiluminescence detection system (Bio-rad, CA, USA) was utilized for protein development. Anti-GAPDH antibody was used to monitor the loading amount. Antibody information was displayed in Supplementary Table 4.

### RNA segmentation and RNA pulldown assays

Human ARST cDNAs (sense and antisense; Biosune biotech, Shanghai, China) and truncated constructs (as follows) were transcribed in vitro using the MEGAscript T7 Kit (Thermo Fisher Scientific, MA, USA). The full-length transcript of ARST was 2116 nt in length; Δ1, Δ2, Δ3, Δ4 and Δ5 corresponded to the 1–470 nt, 471–801 nt, 802–1145 nt, 1146-1477 nt and 1477-2116 nt of the ARST. The mutant ARST (with deletion of 1276-1361 nt) was also constructed for the rescue experiments. For plasmid extraction, OMEGA Endo-free plasmid mini kit II (OMEGA bio-tek, Guangzhou, China) was applied. For restriction enzyme digestion, FastDigest XhoI (Thermo Fisher Scientific, MA, USA) was used and agarose gel electrophoresis images were obtained by the software Quantity One. As for DNA purification and transcription in vitro, TIANquick Mini Purification Kit (Transgen, Beijing, China) and MEGAscript T7 (Thermo Fisher Scientific, MA, USA) were utilized. RNA pulldown assays were performed using a Pierce™ Magnetic RNA-Protein Pulldown Kit (Thermo Fisher Scientific, MA, USA) following the manufacturer’s guideline. Biotinylated ARST was synthesized by Pierce™ RNA 3′ End Desthiobiotinylation Kit (Thermo Fisher Scientific, MA, USA).

### Mass Spectrometry (MS) analysis

Mass spectrometry (MS) analysis was applied to identify the proteins interacting with ARST and its segments, which was performed by Advanced Medical Research Institute of Shandong University. After RNA pulldown process, the protein eluent was preprocessed by Filter Aided Sample Preparation (FASP) method [18]. Subsequently, protein was digested with trypsin (Promega, WI, USA). Peptides were desalted and concentrated using C18-based solid phase extraction prior to analysis by high resolution/high mass accuracy reversed phase (C18) nano-LC-MS/MS. All raw files were processed using Proteindiscover for database searching. MS/MS spectra were searched against the UniProtKB/Swiss-Prot human database. The proteins obtained in the RNA pulldown assay were shown in Supplementary Tables 2.

### Peptide mutation and segmentation assay

For targeting protein mutation and segmentation, ALDOA was mutated at five sites separately: E35D, K42N/R43A, K149A, K294A referring to the former studies [19, 20] (Biosune biotech, Shanghai, China). Based on the binding regions between ALDOA and ARST predicted from the catRAPID database, ALDOA was mutated into two deletion constructs which contained 1-288AA and 78-364AA respectively (Biosune biotech, Shanghai, China). ALDOA and the above mutants were then cloned into the eukaryotic expression vector pcDNA3.1(+) with a C-terminal flag tag separately.

### Co-immunoprecipitation (co-IP) and RNA immunoprecipitation (RIP) assays

A RIP assay was performed using a specific RNA Immunoprecipitation Kit (Geneseed, Guangzhou, China) to detect interaction of the target RNAs and proteins following the manufacturer’s instructions. Whole-cell extracts prepared in the lysis buffer containing protease inhibitor cocktail and RNase inhibitor were incubated on ice for 40 min. The lysate was then centrifuged at 13,000 g, 4 °C for 20 min and the supernatant was cleared by being incubated with Protein A/G magnetic beads (Thermo Fisher Scientific, MA, USA) at 4 °C for 2 h (Magnetic beads were pre-incubated with 5 μg IP-grade antibodies for 30 min at room temperature with rotation about 10 r/min). Liquid after reaction was centrifuged in the spin columns. Eluted by RNase-free water, the dissolved RNA was purified and quantified by qRT-PCR. The proteins of each sample were precipitated with ice-cold acetone for Western blot examination. Specifically, the F-actin antibody (Abcam, Cambridge, UK) was an IgM antibody. Its epitope (antigenic epitope: [4e3. ADL] [21]) targeted on the fibrous actin (F-actin) instead of the globular actin (actin monomer). The anti-IgM magnetic beads (Miltenyi biotec, Bergisch Gladbach, Germany) were also used to complete the cross-linking in the co-immunoprecipitation assay (Supplementary Fig. 4A).

### Lactate test assay

Cells were cultured in 96-well plates, 1 × 10^3^ cells/well for 72 h. The cellular metabolism levels were measured by Lactate assay Kit (Jiancheng Corporation Ltd., Nanjing, China). The lactate in the culture medium was oxidized by lactate dehydrogenase and generated a product that interacted with a probe to produce colors (an absorption maximum at 492 nm) [22].

### Enzymatic activities of PFK, PKM and HK

The U87MG cells were seeded in 6-well plates at 2 × 10^5^ cells/well and cultured for 12 h. Forty-eight hours after transfection, cells were digested by 0.08% EDTA trypsin and the enzyme activities of PFK, PKM and HK were determined by the respective enzyme activity kit (Jiemei Genetech, China) according to the manufacturer’s instructions.

### Immunofluorescence and cytoskeleton co-staining

F-actin Staining Kit-Green Fluorescence-Cytopainter (Abcam, Cambridge, UK) was applied to indicate the relative distribution of actin and other proteins. The cells were seeded on the glass coverslips in 24-well plates and incubated with 1 × Green Fluorescent Phalloidin Conjugate working solution for 1 h [23]. The cells were subsequently incubated with the primary antibodies (anti-DYKDDDK, anti-ALDOA and anti-cofilin) to recognize other proteins overnight followed by the AlexaFluor secondary antibodies for 1 h. The images were captured and handled on Zeiss LSM780 confocal laser scanning microscope system.

### Animal experiments

Lentivirus expression vector aiming at ARST was designed using human eukaryotic translation elongation factor 1 α1 promoter. GL261 cells, U87MG, C57 mice and BALB/c nude mice were used for the experiments in vivo. C57 mice and BALB/c Nude immunodeficiency mice (5 weeks old) were purchased from Beijing Vital River Laboratory Animal Technology Co. Ltd. They were housing in the laboratory animal center of Cheeloo College of Medicine, Shandong University. All the animal experiments were carried out obeying to the Guidelines of Laboratory Animals Using and Caring. The animal experimental procedures were acquired from the admissions of the Animal Care Committee of Cheeloo College of Medicine, Shandong University. After lentivirus infection and puromycin selection, we obtained the experimental cell line LV-EF1a > ARST-CMV > Luciferase/T2A/Puro and the control cell line LV-CMV > Luciferase/T2A/Puro (Cyagen, Guangzhou, China). Mice were anesthetized and the brains regions were stereotactically located, 2 mm away from skull midline of the right frontal area, 2 mm behind the coronal suture. The location was drilled and injected at a depth of 5 mm from the surface of the skull, with a speed of 0.2 μL per 15 s (Total injected 2.5 × 10^6^ cells). After the injection was completed, the needle was left for 2 min and then withdrawn 1 mm every minute. Animals were observed until neurological symptoms or signs appeared, including hunched postures, gait changes, lethargies and weight losses. After implantation for 7d and 14d, the animal imaging system IVIS Spectrum (PerkinElmer, MA, USA) was applied for detecting the variation of the tumor size. Histologic section experiments were performed by the HE staining.

### Reagents and materials

The information of the reagents and antibodies involved in the study are listed in Supplementary Table 4.

### Statistical analysis

GraphPad Prism software (La Jolla, CA, USA) was used for data analysis. ImageJ (National Institutes of Health, MD, USA) was used for figure analysis. T-test was used for comparing the statistical data of two groups. Chi-square test was used to analyze the correlation between the expressions of ARST and the biological features of glioma cells. For qRT-PCR, the experimental systems were repeated three times independently. All data were displayed as mean ± standard error of mean (SEM). *P* value < 0.05 was considered to be statistically significant.

## Results

### LncRNA ARST was significantly downregulated in the glioma samples

**Fig. 1:**
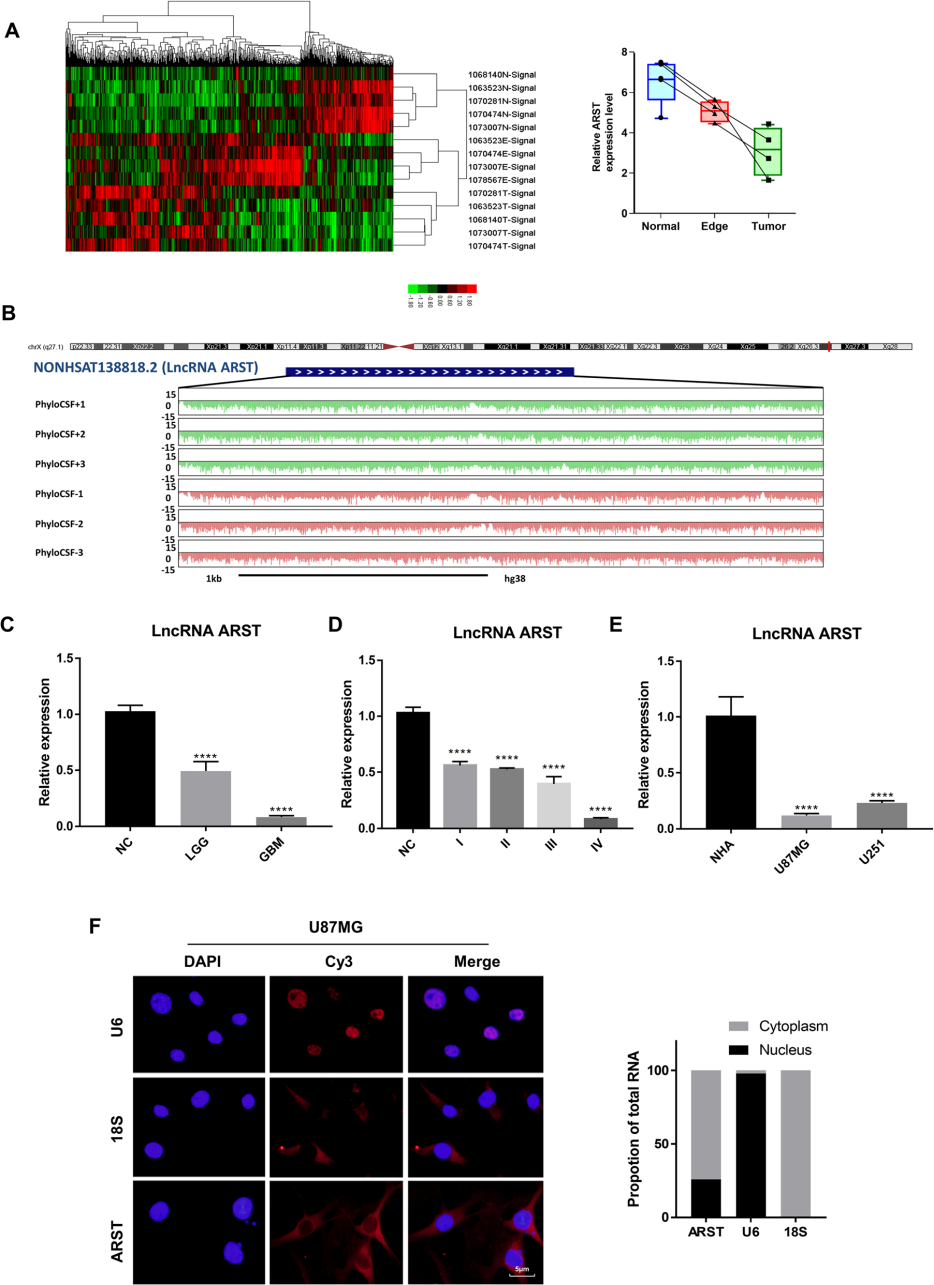
LncRNA ARST was significantly downregulated in the gliomas. **A** The differential expression clusters of lncRNAs in the glioma specimens and paracancerous tissues were shown. The suffix E-Signal represented edge tissues, the suffix T-Signal represented tumor tissues and the suffix N-Signal represented normal tissues. The red color indicated the upregulated lncRNAs and the green color indicated the downregulated lncRNAs (left). The expression levels of ARST in the different tissues were displayed (right). **B** LncRNA location and PhyloCSF value analysis were shown by UCSC genome browser with PhyloCSF data hub. The differential expression levels of ARST in the clinical glioma specimens classified into low grade glioma (LGG), glioblastoma multiforme (GBM) tissues **C** or four World Health Organization (WHO) grades **D** were examined using qRT-PCR. **E** The expressions of ARST in the two glioma cell lines were tested by qRT-PCR compared to normal astrocytes (NHA). Each bar represents mean ± s.d. from three independent experiments. **F** Fluorescence in situ hybridization (FISH) assay was performed to detect the location of ARST in the U87MG cells. Scale bar = 5 μm. Human 18S was used as a cytoplasm internal control and human U6 was used as a nucleus internal control (left). Proportions of ARST and the internal controls were determined in the cytoplasm and nucleus of the cells (right). Data were presented as mean ± s.d. from three independent experiments (****P < 0.001)

To identify novel lncRNAs involved in the development of gliomas, we carried out a lncRNA expression profile analysis using Affymetrix GeneChip® Human Transcriptome Array 2.0. The NONHSAT138818.2, which we named lncRNA ARST (ALDOA related specific transcript) exhibited significant downregulation in the glioma tissues compared to the paracancerous and normal tissues (Fig. 1A). Then we used NCBI ORFinder online tool (https://www.ncbi.nlm.nih.gov/orffinder/) to detect the number of ORFs in this RNA sequence. The results showed that 23 sense ORFs with potential to encode peptides were included. However, these 23 peptides indicated no significantly similar proteins or peptides when they were compared with the Swiss-Prot database using the NCBI BLAST tool (Supplementary Table 1). Additionally, the PhyloCSF value of ARST, which was calculated to verify the conservation of the sequence, was minus. In conclusion, we identified that ARST could not encode protein and it was a long non-coding RNA [24, 25] (Fig. 1B).

We subsequently analyzed the expression levels of ARST in more glioma and corresponding non-cancerous tissues using qRT-PCR analysis. The results showed that the expressions of ARST were significantly downregulated in the glioblastoma multiforme (GBM) tissues compared to the low grade glioma (LGG) and non-cancerous tissues (Fig. 1C). Furthermore, ARST levels were negatively correlated with the pathological grades of the gliomas. (Fig. 1D). In the glioblastoma cell lines U87MG and U251, ARST was considerably lower in comparative to normal human astrocytes (Fig. 1E), further suggesting the potential role of ARST in gliomagenesis.

According to the NONCODE database, ARST is one of the transcripts derived from LINC00632, which mainly distributes in the human brain based on the GEPIA database (Supplementary Fig. 1A). Compared with normal tissues, the expressions of LINC00632 were significantly downregulated in both tissues of GBM and LGG (Supplementary Fig. 1B-D). Moreover, low LINC00632 expression correlated with poor overall survival and disease-free survival in the glioma patients (Supplementary Fig. 1E, F). Fluorescence in situ hybridization (FISH) assay was further utilized to examine the distribution of ARST in the U87MG cells. The results showed that ARST mainly located in the cytoplasm of the cells (Fig. 1F). The U251 cell line displayed a similar result (Supplementary Fig. 1G).

### ARST inhibited the malignant phenotypes of glioma cells

**Fig. 2:**
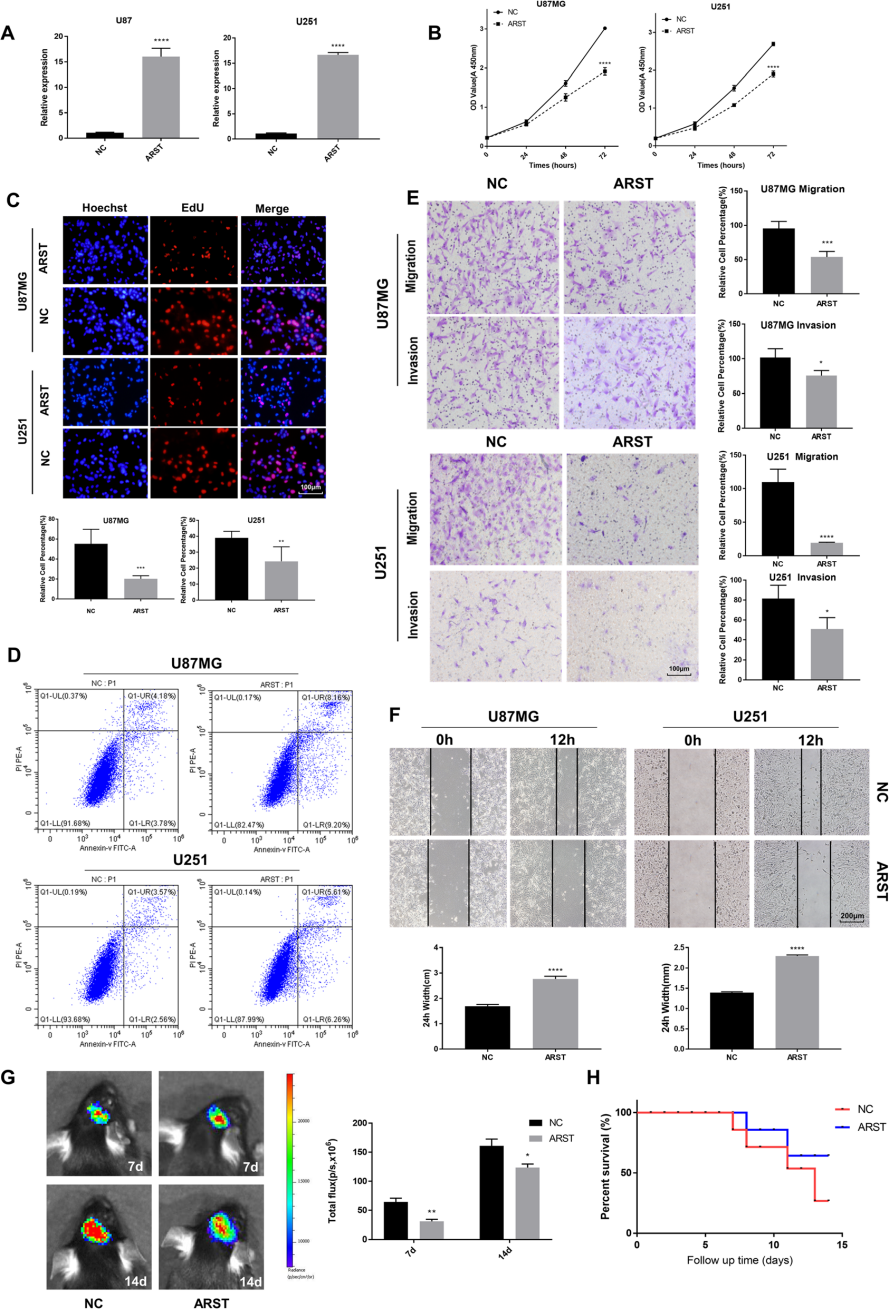
ARST inhibited the malignant phenotypes of the glioma cells. **A** The efficiencies of ARST overexpression in the U87MG and U251 cells were tested by qRT-PCR. **B** The growth curves of the transfected glioma cells were shown using CCK-8 assay. **C** The EdU staining assay was performed to determine the proliferation of the transfected U87MG and U251 cells. Scale bar = 100 μm. **D** Apoptosis of the transfected cells were analyzed by staining of Annexin V and PI followed by flow cytometry assay. **E** Migration and invasion of the transfected cells were determined by transwell assay. Scale bar = 100 μm. **F** Wound-healing assay was performed. Scale bar = 200 μm. **G** Representative images and total flux of the C57 mice 7 days and 14 days after intracranial implantation of the GL261 cells transfected with luciferase tagged ARST or control plasmids by IVIS spectrum. **H** Surviving curves of the C57 mice with xenografted tumors intracranially was recorded for 15 days. All data were presented as mean ± s.d. from three independent experiments (*P < 0.05, **P < 0.01, ***P < 0.005, ****P < 0.001)

To elucidate the functions of ARST in gliomas, we transfected both the U87MG and U251 glioma cells with the plasmids expressing ARST (oeARST) or ARST Smart Silencer (siARST). Nonspecific vectors were used as the negative control. qRT-PCR analysis indicated an increase by approximately 15 times in the oeARST-transfected cells compared with the control (Fig. 2A). However, siARST only resulted in a modest decrease of ARST in these cells (Supplementary Fig. 2A, B).

A traditional CCK-8 assay was performed to evaluate whether ARST would affect viability of the glioma cells. As the results shown in Fig. 2B, cell growth was markedly inhibited in both of the U87MG and U251 cells when ARST was overexpressed (Fig. 2B). However, the change in cell growth caused by knocking down ARST was not significant (Supplementary Fig. 2C, D). To further clarify whether the reduction of cell viability in ARST upregulated cells resulted from the changes in cell proliferation or apoptosis, EdU cell proliferation assay was applied. As depicted in Fig. 2C, the number of EdU positive cells was significantly decreased when ARST was overexpressed in both cell lines (Fig. 2C). What’s more, staining with Annexin V and PI followed by flow cytometry assay indicated that 17.36% of U87MG and 11.87% of U251 cells underwent apoptosis with the upregulation of ARST; while only 7.96 and 6.13% of apoptotic cells were observed in the cells transfected with the negative control (Fig. 2D).

As one of the most aggressive malignant brain tumors, gliomas are characterized with massive infiltrative growth without boundary from surrounding tissues [26]. Therefore, to investigate whether ARST contributes to the migration and invasion of glioma cells, transwell and wound-healing assays were performed. The results showed that number of the migrating or invading cells with ARST overexpression was decreased compared to that of the control cells, with average reduction rate of 34.5% for the U87MG cells and 39.9% for the U251 cells, respectively (Fig. 2E). On the other hand, the recovery of scratches was significantly suppressed when ARST was upregulated in the both cells line after 12 h incubation (Fig. 2F) [27].

Considering the in vitro involvement of ARST in glioma cell proliferation, migration and invasion, we extended this study to determine the impact of ARST on tumorigenic capabilities of gliomas in vivo. When the GL261 cells infected with lentiviral vectors expressing ARST or negative control were intracranially implanted into the C57 mice, we observed a significant decrease in tumor formation in the tumor-bearing mice when ARST was overexpressed (Fig. 2G). Furthermore, these mice achieved longer survival in comparison to the control mice (Fig. 2H).

### ARST interacted with ALDOA to exert its functions in glioma development

**Fig. 3:**
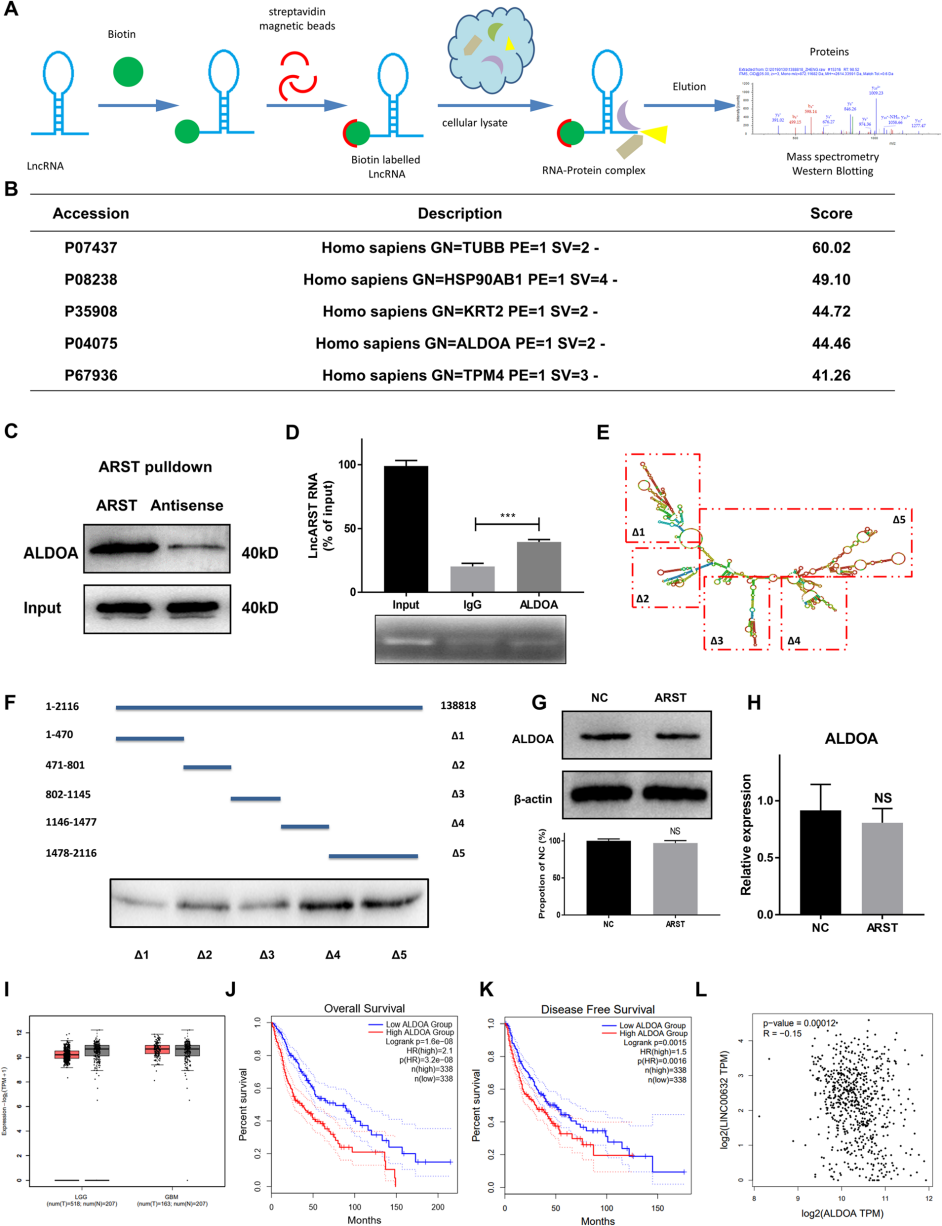
LncRNA ARST interacted with ALDOA to exert its functions in glioma development. **A** Schematic illustration of RNA pulldown followed by mass spectrometry assay to detect potential proteins interacting with ARST. **B** Potential binding proteins were listed based on the score of peptide identification from mass spectrometry assay. **C** ALDOA was detected by Western blot from the eluted proteins following RNA pulldown assay using biotinylated sense and antisense strands of ARST. **D** RIP assay was performed with extracts of U87MG cells using anti-ALDOA or mouse IgG. IgG served as the negative control. ARST enriched in anti-ALDOA and IgG pull-downs was determined relative to the input. **E** Schematic diagram of truncated biotin-labeled ARST RNA probe **F** RNA pulldown followed by western blot was performed to detect the binding affinity of truncated ARST with ALDOA. Western blot **G** and qRT-PCR **H** were performed to detect the expression levels of ALDOA in the ARST overexpressed cells compared to the control. **I** Graphic representation of relative ALDOA expression levels (TPM) in different tissues (GBM vs. GTEx, LGG vs. GTEx). GBM and LGG represented glioblastoma multiforme and low grade gliomas in the TCGA datasets. GTEx represented normal brain tissues in the GTEx database. **J** Overall and **K** disease free survivals of the glioma patients with relative low or high level of ALDOA expressions were assessed (cut-off value is 50%). **L** The correlation of the expressions between ALDOA and LINC00632 were displayed based on the TCGA database. Data were presented as the mean ± s.d. from three independent experiments (***P < 0.005)

In order to identify the proteins that interact with ARST, biotin-labeled RNA pulldown followed by a mass spectrometry-based assay was conducted (Fig. 3A). The results revealed that 125 proteins were differentially bound to the sense and anti-sense strands of ARST in the U87MG cells (Supplementary Table 2). Based on the score of peptide identification, we discovered that ALDOA was one of the highly enriched proteins (Fig. 3B), which was further verified by Western blot (Fig. 3C). Moreover, in the silver staining assay, coinciding with the position of ALDOA, a 40 kD band showed up in the protein lysate that bound to the sense strand of ARST, while it was missing in the lysate that bound to the anti-sense strand of ARST (Supplementary Fig. 3A). To further validate the physical interaction between ARST and ALDOA, we subsequently performed a RIP assay with an anti-ALDOA antibody and found that ARST was obviously enriched (Fig. 3D), evidently confirming the above results.

To determine the specific fragments of ARST required for its interaction with ALDOA, we constructed a series of ARST truncation plasmids according to the loop structures of ARST transcript (Fig. 3E) and conducted RNA pulldown assay. The results indicated that the 1146-1477 nt (Δ4) and 1478-2116 nt (Δ5) regions of ARST potentially mediated its interaction with ALDOA (Fig. 3F). However, overexpression of ARST affected neither the protein or mRNA levels of ALDOA (Fig. 3G, H). These results suggested that ARST might exert its functions via binding to ALDOA, instead of regulating the expression of ALDOA.

We subsequently examined the transcriptional and survival data of ALDOA in patients with LGG and GBM from the GEPIA database. The results showed that there was no significant difference in the expression levels of ALDOA between the LGG/GBM and normal brain tissues (Fig. 3I). However, the overall and disease-free survivals of the glioma patients with high level of ALDOA were obviously lower than that with low level of expression (Fig. 3J, K). Then we took a step further to explore the correlation of LINC00632 and ALDOA via log2 FPKM. The Pearson’s correlation co-efficiency of LINC00632 and ALDOA was − 0.15 (Fig. 3L), which might further indicate that the relationship between ARST and ALDOA did not occur on the level of transcription.

### ARST regulated ALDOA mediated actin filament integrity instead of its enzymatic activity

**Fig. 4:**
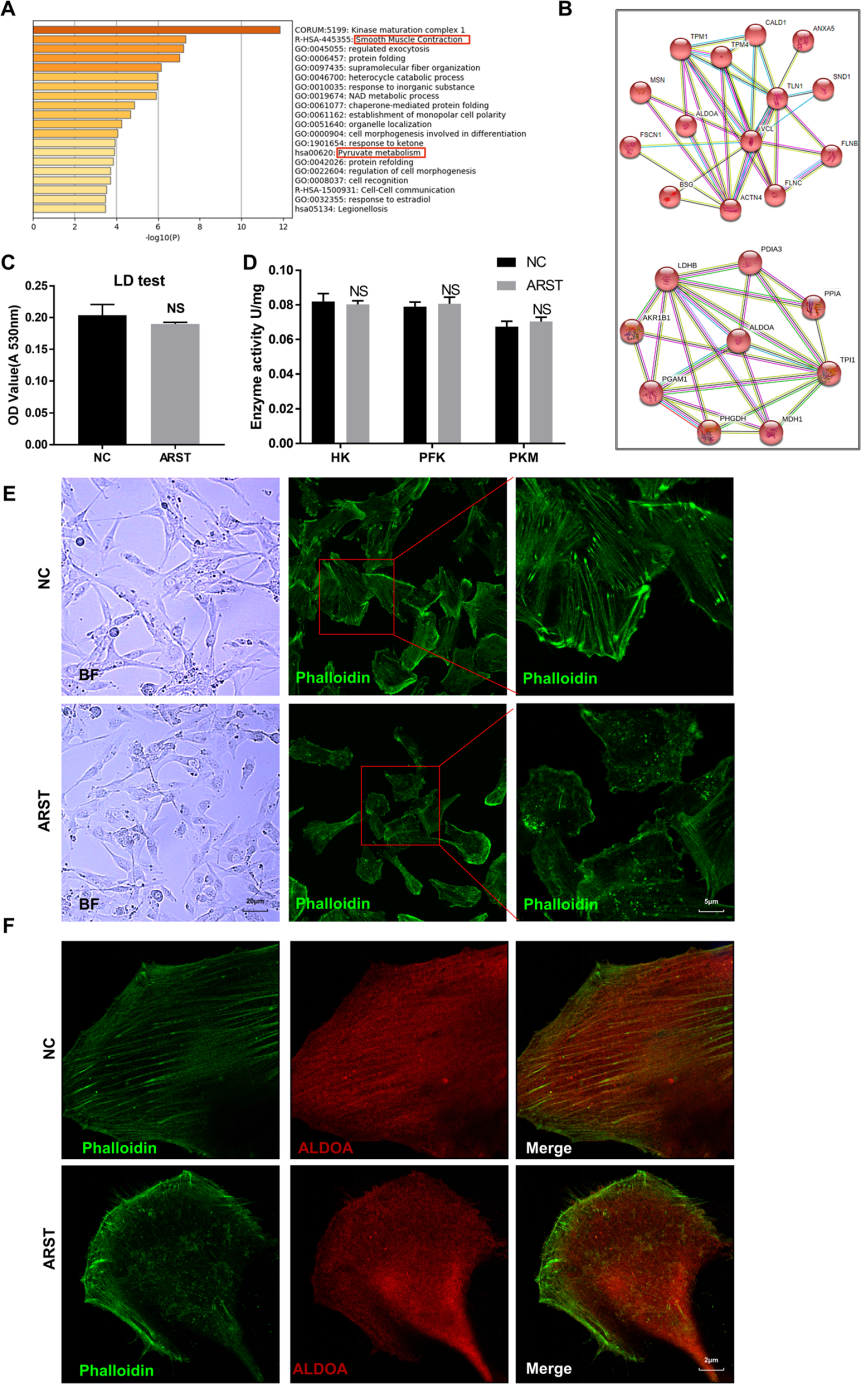
ARST regulated ALDOA mediated actin filament integrity instead of its enzymatic activity. Metascape enrichment analysis **A** and PPI network **B** for the list of proteins that specifically bound to the full-length ARST sense RNA probe. **C** Lactate levels were measured in the U87MG cells upregulated with ARST (ARST) compared to the negative control (NC). **D** Enzymatic activities of HK, PKM and PFK were determined in the indicated cells. **E** Phase contrast (Scale bar = 20 μm) and confocal microscopes (Scale bar = 5 μm) were utilized to observe the morphological changes of the transfected cells and the phalloidin stained F-actin cytoskeleton in them. **F** Co-staining of ALDOA and F-actin in the indicated cells was performed. Scale bar = 2 μm. All data were presented as mean ± s.d. from three independent experiments

Subsequently, we analyzed the proteins obtained in RNA pulldown by mass spectrometry. The proteins that specifically bound to the sense strand of ARST were used to construct a PPI (Protein-protein interaction) network using the STRING database (https://www.string.org) (Supplementary Fig. 3B). At the same time, these proteins were enriched by the Metascape database (www.metascape.org) and clustered using its built-in MCODE algorithm (Fig. 4A, B). We found that the pathways in which ALDOA was closely involved were tumor metabolism and smooth muscle contraction. According to the description of smooth muscle contraction in the Reactome database (https://reactome.org), this pathway mainly includes cytoskeleton related protein components such as actin, myosin and intermediate filaments. As previously reported, this refers to the non-enzymatic or moonlight effect of ALDOA [11].

To determine whether ARST regulated the enzymatic activity of ARST, the concentration of lactate in the culture medium of U87MG cells was examined. As the results shown in Fig. 4C, no significant fluctuation appeared in the lactate levels when ARST was overexpressed (Fig. 4C). Furthermore, the activities of the key enzymes involved in glycolysis had hardly changed in these cells (Fig. 4D).

On the other hand, we noticed an obvious morphological change in the ARST upregulated cells which exhibited less dendrite-like structures in comparison to the control cells. Furthermore, immunofluorescence with phalloidin staining indicated that the actin stress fibers were intact in the control cells, however, they almost disappeared in the cells with ARST overexpression (Fig. 4E). In order to clarify the relationship between ALDOA and F-actin, dual staining with the antibody to recognize ALDOA as well as phalloidin was performed. The result showed that in the control cells, ALDOA exhibited a significant enrichment on the actin filaments. In contrast, with the disappearance of actin fibers, ALDOA were diffusely distributed in the ARST upregulated cells (Fig. 4F). This phenomenon evidently suggested that ARST might regulate the role of ALDOA in actin fiber integrity instead of its enzymatic activity.

### ARST decreased the interactions of ALDOA, cofilin and F-actin, respectively

Considering the negative role of ARST in ALDOA mediated actin fiber integrity, we made a step further to explore whether other proteins were involved in the depolymerization of actin filaments when ARST was overexpressed. Based on the PDB database and previous literature [28], we found that cofilin, an actin depolymerizing protein (ADP) took effects in interacting with F-actin and further depolymerizing it. A subsequent separation from cofilin leads to the release of G-actin monomers, which would be further used to promote the assembly of new F-actin fiber [14]. Most interestingly, ALDOA and cofilin shared an extensive overlap of binding sites on F-actin [11, 28, 29]. It was therefore proposed that ARST detached ALDOA from F-actin. The exposed binding sites were further bound by cofilin, which led to the depolymerization of actin filaments.

**Fig. 5:**
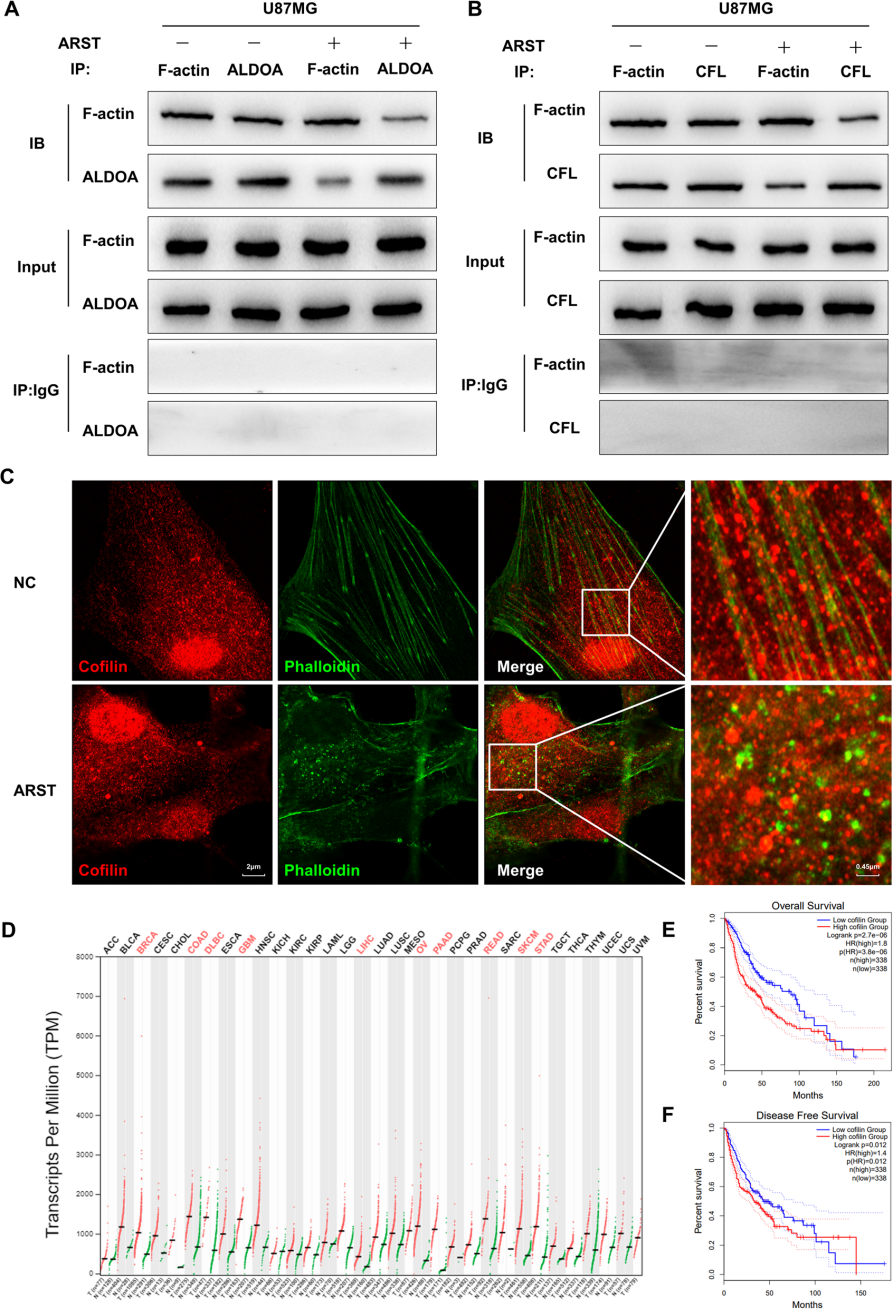
ARST decreased the interactions of ALDOA and cofilin with F-actin, respectively. **A, B** Co-immunoprecipitation assay was performed to detect the binding capacity of F-actin and ALDOA or cofilin in the U87MG cells transfected with ARST or negative control vector. **C** Co-staining of cofilin and phalloidin labelled F-actin cytoskeleton was performed in the transfected cells. Images were taken under confocal microscope. Scale bar = 2 μm (left) / 0.45 μm (right). **D** Transcripts per million (TPM) of cofilin in different cancers based on the TCGA database. **E** Overall and **F** disease free survivals of the glioma patients with relative low or high level of cofilin expressions were assessed in the GEPIA database (cut-off value is 50%)

To prove the above hypothesis, co-immunoprecipitation was performed to detect the interactions between ALDOA, cofilin and F-actin. The results demonstrated that upregulation of ARST significantly decreased the binding capacity of ALDOA to F-actin (Fig. 5A). The interactions between cofilin and F-actin were reduced as well, compared to the negative control (Fig. 5B). Immunofluorescence of cofilin as well as phalloidin was further conducted. The result showed that in the control cells, cofilin localized as obvious dotted structures on the actin stress fibers. Overexpression of ARST led to obvious disappearance of F-actin. More interestingly, the distributions of cofilin and depolymerized actin were separated in the cytoplasm, in line with the role of cofilin in releasing G-actin monomers upon the depolymerization of actin filaments (Fig. 5C). Overall, these phenomena evidently suggested that cofilin might be involved in the depolymerization of actin stress fibers following the detachment of ALDOA from F-actin by upregulation of ARST.

According to previous literatures, the phosphorylation site of cofilin localized in the actin-binding domain and inhibited its binding to actin filaments, blocking cofilin ability to the promotion of filament disassembling [30]. Therefore, we made a step further to investigate whether upregulation of ARST altered the phosphorylation status of cofilin and regulated the signaling pathway involved. However, the results of qRT-PCR demonstrated that the expression levels of RhoA, ROCK1, LIMK1 and cofilin did not change significantly when ARST was overexpressed. Moreover, the protein levels of ROCK1, p-LIMK1, LIMK1, p-cofilin and cofilin had hardly changed in the ARST up-regulatged cells based on the results of immunoblotting analysis (Supplementary Fig. 4B, C), indicating that even though RhoA/ROCK1/LIMK/cofilin pathway was closely related to cofilin-related cytoskeleton dynamics and cell migration, it was not involved in the regulatory process of ARST.

We subsequently examined the transcriptional and survival data of cofilin in the patients with LGG and GBM from the GEPIA database. The results showed that the expressions of cofilin were significantly upregulated in the LGG/GBM samples compared to the normal brain tissues (Fig. 5D). Consistently, the overall and disease-free survivals of the glioma patients with high-levels of cofilin were lower than that with low level expressions of cofilin (Fig. 5E, F).

### ARST interacted with the binding sites of ALDOA and F-actin

**Fig. 6:**
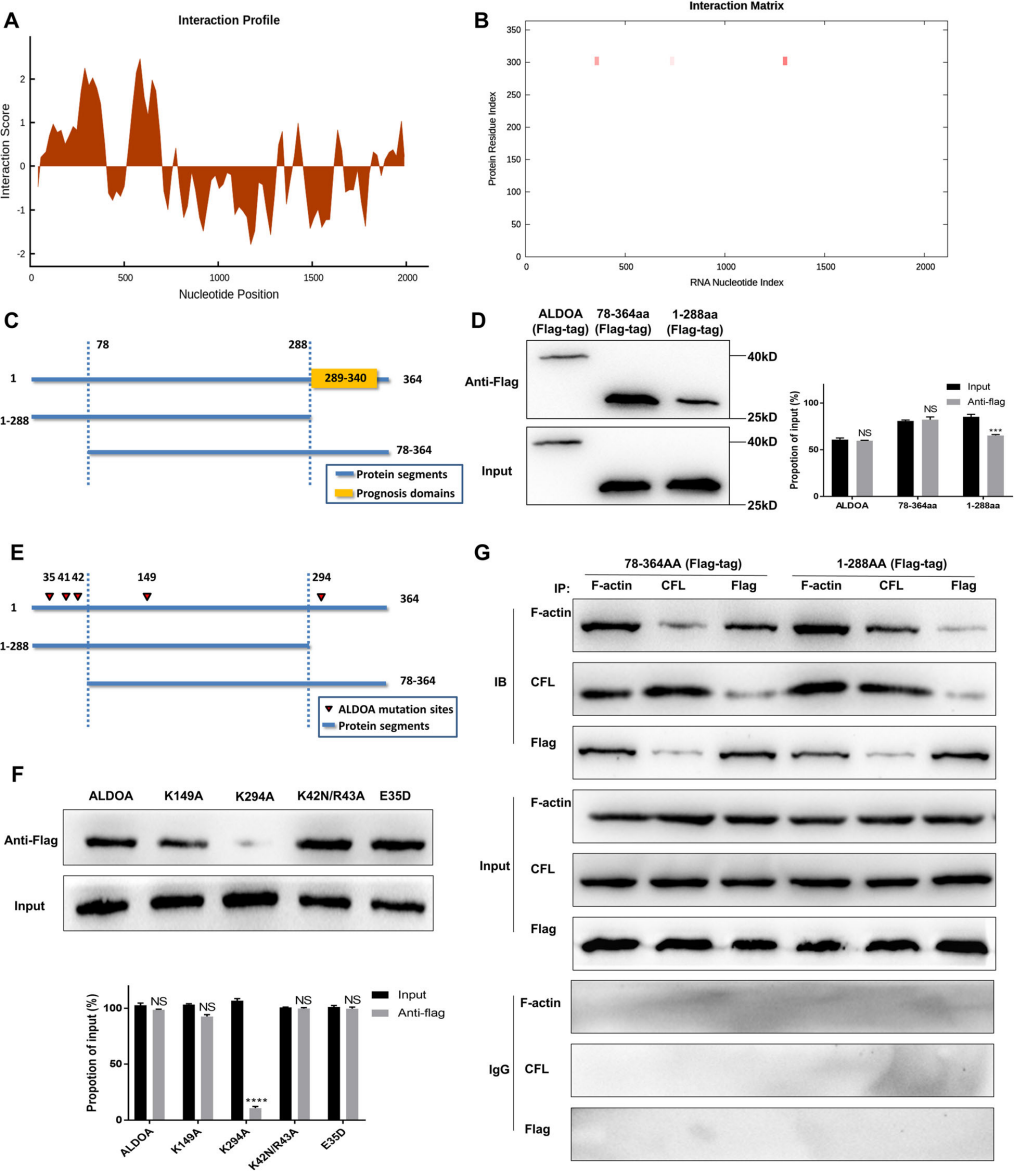
ARST interacted with the binding sites of ALDOA and F-actin. **A, B** The catRAPID database was referenced to predict the binding domains of ARST and ALDOA. **C** Truncated ARST was demonstrated by the diagrammatic sketch. The binding domain predicted by catRAPID was highlighted in yellow. **D** RNA pulldown followed by western blot was performed to detect the binding capacity of truncated ALDOA with ARST. **E** The mutation sites on ARST, highlighted by red arrows, were demonstrated by diagrammatic sketch. **F** RNA pulldown followed by western blot was performed to detect the interactive capacities of different mutant ALDOA with ARST. **G** Co-immunoprecipitation assay was performed to detect the binding capacity of F-actin and cofilin in the presence of ALDOA mutants. All results were presented as mean ± s.d. from three independent experiments (****P < 0.001)

In order to investigate the detailed mechanisms involved in ARST mediated separation of ALDOA from F-actin, catRAPID database (http://service.tartaglialab.com) was utilized to predict the binding sites of ARST on ALDOA. The results demonstrated that ARST potentially bound to the 289-340AA of ALDOA (Fig. 6A, B). On the other hand, ALDOA potentially interacted with the 1276-1361 nt of ARST, which was consistent with the previous result in Fig. 3F (Supplementary Fig. 5A). To verify the specific regions of ALDOA required for its binding with ARST, we constructed ALDOA truncation plasmids including or excluding the predictive domain based on the catRAPID database and conducted RNA pulldown assay (Supplementary Table 3). The results indicated that the 289–364AA of ALDOA was indeed required for its interaction with ARST (Fig. 6C, D).

What’s more, it was previously reported that the amino acids E35, K42, R43, K149, K294 of ALDOA contributed to the interaction between ALDOA and actin filaments [15, 20]. To verify whether ARST affected any of the above amino acids, we constructed a series of ALDOA plasmids with the mutated amino acids E35D, K42N/R43A, K149A and K294A respectively (Fig. 6E; Supplementary Fig. 5B-E). The subsequent RNA pulldown assay indicated that K294 was essential for the interaction between ARST and ALDOA (Fig. 6F). It was thus hypothesized that ARST might bind to K294 of ALDOA, which in turn weakened the interaction of ALDOA with F-actin.

As mentioned above, ALDOA and cofilin shared an extensive overlap of binding sites on F-actin. To investigate whether deletion of the binding sites between F-actin and ALDOA by introducing ALDOA deletion mutants affected the interaction between F-actin and cofilin, we transfected the ALDOA truncated plasmids including or excluding the binding domain of ALDOA and F-actin, and subsequently repeated the co-immunoprecipitation to detect the interaction between cofilin and actin filaments. The results showed that overexpressing the 289–364AA of ALDOA significantly decreased the binding capacity of cofilin to F-actin. In contrast, the 1–288AA of ALDOA did not have such an effect, evidently confirming the involvement of cofilin in this process (Fig. 6G).

### Upregulation of ALDOA alleviated the inhibitory effects of ARST in gliomas

**Fig. 7:**
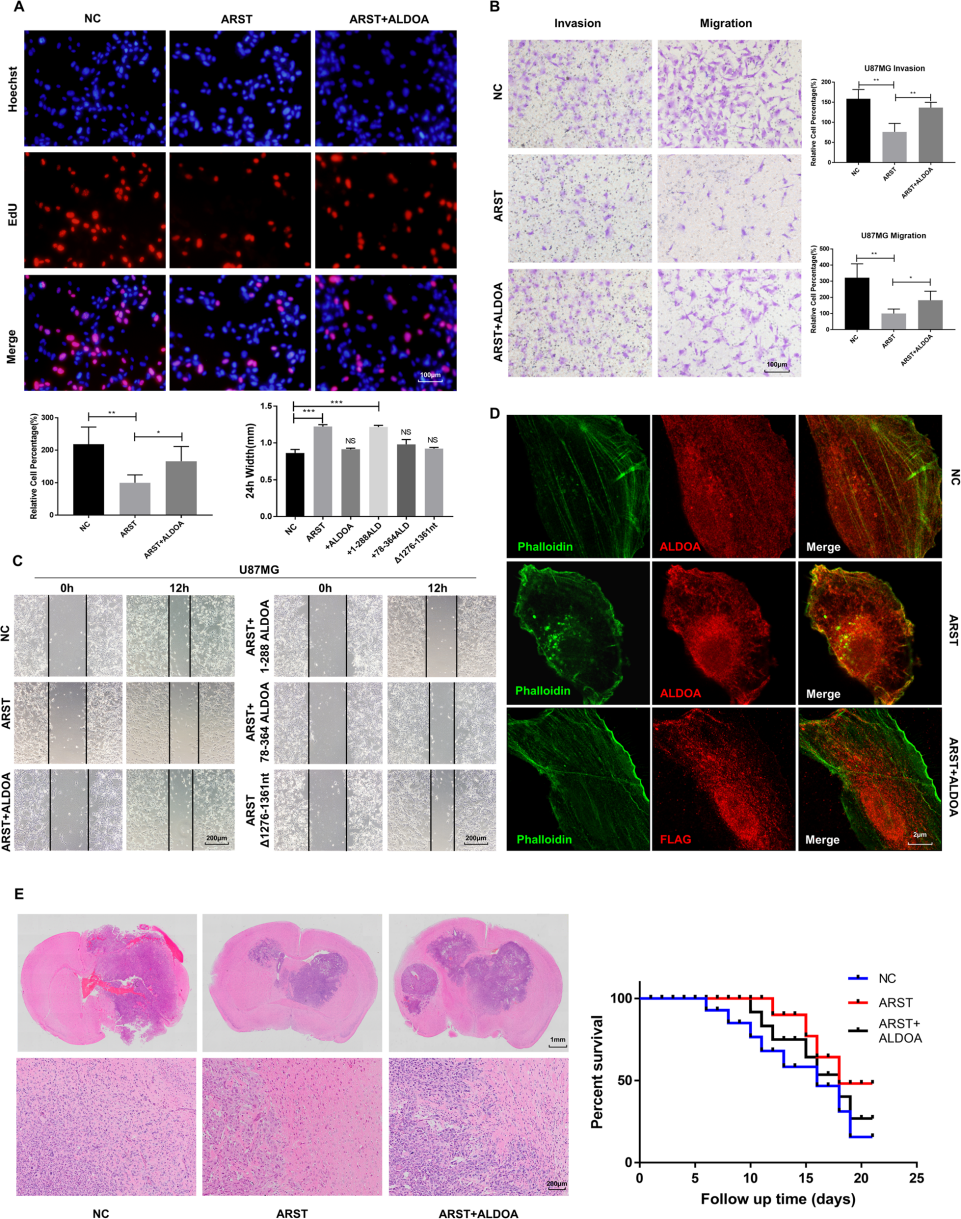
Upregulation of ALDOA alleviated the inhibitory effects of ARST in gliomas. The U87MG and U251 cells were transfected with the plasmids expressing empty vector (NC), ARST together with empty vector (ARST+vector), ARST together with ALDOA (ARST+ALDOA), ARST together with 1-288AA ALDOA (ARST+ 1-288ALDOA), ARST together with 78-364AA ALDOA (ARST+ 78-364ALDOA) or ARST lack of 1276-1361 nt (ARST Δ1276-1361 nt). **A** EdU assay was performed to detect the proliferation of the indicated cells. Scale bar = 100 μm. **B** Migration and invasion of the transfected cells were determined by transwell assay. Scale bar = 100 μm. **C** Wound-healing assay was performed to detect the migratory abilities of the indicated cells. Scale bar = 200 μm. **D** Confocal microscope was utilized to observe the co-staining of ALDOA and phalloidin labelled F-actin cytoskeleton in the indicated cells. Scale bar = 2 μm. **E** Representative micrographs of HE-stained sections of mouse brain tissues under low- (Scale bar = 1 cm) and high- (Scale bar = 200 μm) power magnifications 15 days after intracranial implantation of the U87MG cells infected with a lentiviral vector expressing NC, ARST or ARST+ALDOA. Curves show the survival rates of the xenografted mice. All results were presented as mean ± s.d. from three independent experiments (*P < 0.05, **P < 0.01, ***P < 0.005, ****P < 0.001)

Given that ARST suppressed gliomagenesis and ALDOA was a potential target of ARST, we next investigated whether ALDOA represented a functional link for the biological changes observed in the glioma cells with ARST upregulation. To confirm this hypothesis, U87MG and U251 cell lines were first transfected with the plasmids overexpressing ARST, which were termed oeARST cells. Furthermore, we transfected these oeARST cells with the plasmids designated to ALDOA to investigate the responses to ALDOA upregulation. As expected, the efficiency of ARST in inhibiting cell proliferation (Fig. 7A), invasion and migration (Fig. 7B) were significantly alleviated when ALDOA was induced. Considering that the 289-364AA of ALDOA and 1276-1361 nt of ARST were required for their physical interaction. The rescue experiments with relative deletion mutants were conducted. The results of wound-healing assay showed that the ALDOA mutant lack of 289-364AA could not rescue the decreased migration of the oeARST cells compared to the wild type ALDOA. On the other hand, the ARST mutant lack of 1276-1361 nt was unable to inhibit the migration of the oeARST cells in comparison to the wild type ARST (Fig. 7C). Immunofluorescence analysis was additionally applied. The results showed that upregulation of ALDOA did significantly restore the actin stress fibers in the oeARST cells (Fig. 7D). What’s more, the nude mice which were intracranially transplanted with the ARST/ALDOA double upregulated cells exhibited larger tumor size and poorer prognosis compared to that transplanted with oeARST cells. Under a high power field of view, the beneficial effect of oeARST in inhibiting the glioma cells from invading into the paracancerous tissues was significantly compromised when ALDOA was simultaneously induced (Fig. 7E). Taken together, all these data suggested that the tumor suppressive role of ARST in gliomagenesis might be largely dependent on ALDOA.

## Discussion

Glioma is one of the most common malignant tumors in the central nervous system. It is characterized with rapid progression, poor prognosis and high recurrence rate. Due to the strong infiltrative ability to the surrounding tissues, the recurrence rate of the glioma is almost 100%, making it extremely difficult for neurosurgeons to deal with it effectively [31].

Cytoskeleton plays an essential role in the motion dynamics of the cells [32], and one of the most important components of which is fibrous actin (F-actin). Marie France Carlier et al. reported that actin filaments were depolymerized by cofilin, a kind of actin depolymerization protein (ADP) [14]. It was conducive to the fracture of the mature actin fibers and accelerated the formation of new F-actin. On the other hand, Yu Chan Chang et al. elaborated the important role of ALDOA in the polymerization and stabilization of actin filaments [15]. Therefore, under normal circumstances, the polymerization and depolymerization of F-actin exist together, which maintains a dynamic balance to ensure the normal movement, morphological changes and other life activities of the cells.

Recently, some researchers found that ALDOA and cofilin had overlapping binding sites on F-actin [11]. Among them, ALDOA, which was indispensable for actin fiber reorganization, had a stronger capacity to bind F-actin, so that the actin cytoskeleton was kept in a stable state in most cases. Compared with the cells in normal tissues, ALDOA and cofilin were often overexpressed in the cells of various tumors. Especially in the glioma cells, which exhibited a strong invasive ability, cofilin was significantly upregulated [33, 34]. High level of cofilin led to the enhanced depolymerization and re-polymerization of actin fibers which promoted malignant invasion and migration of gliomas [35]. Furthermore, it was also reported that both ALDOA and cofilin could be used as promising indicators for glioma radio-sensibility and prognosis [36].

Up to now, researches in tumor development have focused mostly on protein-coding genes. However, the functions and contributions of non-coding RNAs remain insufficiently defined [16]. Based on a large-scale microarray profiling, we identified a novel long non-coding RNA, ALDOA Related Specific Transcript (ARST) which was significantly suppressed in the glioma tissues. In contrast, we found the information of the parental transcript of ARST, LINC00632. According to the TCGA database, LINC00632 is downregulated in the glioma tissues and glioma cell lines. The glioma patients with high expression level of LINC00632 are associated with better long-term and disease-free survivals, potentially suggesting a similar role of ARST in gliomagenesis.

To verify the assumption, we overexpressed ARST in the glioma cell lines. The results showed that ARST inhibited the malignant phenotypes of the glioma cells such as viability, proliferation, invasion and migration. However, the level of lactate and activities of the key enzymes involved in glycolysis had hardly changed, suggesting a non-metabolic role of ARST in these cells. We further identified that ARST interacted with the crucial domain of ALDOA through which ALDOA binds to F-actin. Overexpression of ARST decreased the binding capacities of ALDOA to F-actin, leading to the severe disintegration of actin fibers. The interaction between cofilin and actin was affected as well.

**Fig. 8:**
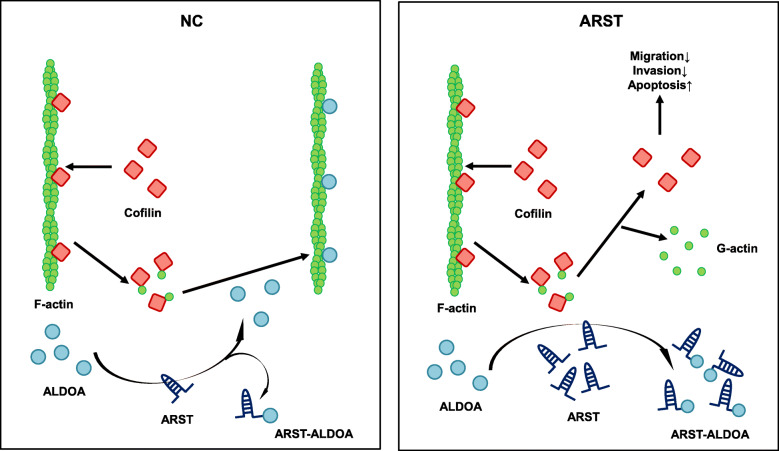
Schematic model of ARST-mediated F-actin cytoskeleton modulation in gliomas

It is thus hypothesized that in normal cells, the polymerization and depolymerization of F-actin are orderly and strictly controlled, among which ARST plays a negative regulatory role in occupying ALDOA so that the excessive formation of F-actin is avoided. However, in the glioma cells, downregulation of ARST decreases its restraint on ALDOA. Simultaneous induced expression of cofilin accelerates the speed of depolymerization of old F-actin and re-polymerization of new actin fibers. That probably explains the significant infiltrative property of gliomas. When we overexpressed ARST in the glioma cells, it interrupted the interaction of ALDOA and actin filaments, so that more binding sites of F-actin were exposed to cofilin, which in turn led to the depolymerization of actin cytoskeleton. On the other hand, without the interaction of ALDOA, formation of new actin fibers were significantly inhibited (Fig. 8).

Taken together, it is concluded that ARST performs its function via regulating the dynamic equilibrium and integrity of actin cytoskeleton through ALDOA and cofilin, which in turn modifies the morphology and invasive properties of the glioma cells. This has added new perspective to its role in tumorigenesis, thus providing potential therapeutic targets for glioma treatment.

## Conclusions

In this study, we reported that a novel lncRNA ARST was downregulated in the gliomas. Overexpression of ARST in the glioma cells significantly suppressed cell viability, proliferation, invasion and migration in addition to promotion of apoptosis. The tumorigenic capacity of these cells in vivo was reduced as well. We further demonstrated that the tumor suppressive effects of ARST could be mediated by a direct binding to ALDOA, which together with cofilin, maintains an orderly and strictly controlled balance of polymerization and depolymerization of actin filaments. Upregulation of ARST interrupted the interaction of ALDOA and actin fibers, so that more binding sites of F-actin were exposed to cofilin, which in turn led to the depolymerization of actin cytoskeleton. On the other hand, without the interaction of ALDOA, formation of new actin fibers was significantly inhibited.

ARST performed its function via regulating the dynamic equilibrium and integrity of actin cytoskeleton through ALDOA and cofilin, which in turn modified the morphology and invasive property of the glioma cells. This has added a new perspective to its role in tumorigenesis, thus providing a potential target for glioma diagnoses, therapies, and prognoses.

## Supplementary Information

**Additional file 1: Figure S1.** (A) The expressions of LINC00632 in normal people (left) and glioma patients (right) based on the TCGA database. (B) The differential expression levels of LINC00632 are displayed in different tumors compared with the respective normal tissues. (C) Graphic representation of relative LINC00632 expression level (TPM) in different tissues (GBM vs. GTEx, LGG vs. GTEx). GBM and LGG represented glioblastoma multiforme and low grade gliomas in the TCGA datasets. GTEx represented normal brain tissue in the GTEx database. (D) Transcripts per million (TPM) of LINC00632 in different cancers according to the TCGA database. GBM and LGG are highlighted in green (downregulated). (E) Overall and (F) disease free survivals of the glioma patients with relative low or high level of LINC00632 expressions were assessed in the GEPIA database (cut-off value is 50%). (G) Fluorescence in situ hybridization (FISH) assay was performed to detect the location of ARST in the U251 cells. Human 18S was used as a cytoplasm internal control and human U6 was used as a nucleus internal control. Proportions of ARST and the internal controls were determined in the cytoplasm and nucleus of the cells. Scale bar = 5 μm.

**Additional file 2: Figure S2.** (A) The efficiencies of ARST knockdown in the U87MG and U251 cells were tested by qRT-PCR. **P < 0.01. (B) The growth curves of the transfected glioma cells were determined using CCK-8 assay. (C) Lentivirus infected GL261 cells together with GFP and luciferase were examined under immunofluorescent microscope. Scale bar = 100 μm. All results were represented mean ± s.d. from three independent experiments.

**Additional file 3: Figure S3.** (A) Silver staining assay was performed to detect the eluted proteins following RNA pulldown assay using biotinylated sense and antisense strands of ARST. (B) The proteins that only bound to the sense strand of ARST were used to construct a PPI (Protein-protein interaction) network using the STRING database.

**Additional file 4: Figure S4.** (A) The schematic diagram of F-actin immunoprecipitation. (B) Western blot analysis showed the changes of phosphorylation status of cofilin and LIMK1 in the U87MG cells after upregulation of ARST. GAPDH was used as the internal control. (C) qRT-PCR analysis was performed to show the mRNA levels of RhoA/ROCK1/LIMK/cofilin in the U87MG cells when ARST was overexpressed.

**Additional file 5: Figure S5.** (A) The catRAPID online database was utilized to predict the binding domains of ALDOA and ARST. Interaction propensities of different regions were shown. (B-E) the specific sites of ALDOA that were mutated in the study were demonstrated.

**Additional file 6: Figure S6** The original blot images in the manuscript, which corresponded to Fig. 3C (A), Fig. 3F (B),Fig. 3G (C) and Fig. 5A and B (D).

**Additional file 7: Figure S7.** The original blot images in the manuscript, which corresponded to Fig. 6D (A), Fig. 6F (B) and Fig. 6G (C).

**Additional file 8: Figure S8** The original blot images in the manuscript, which corresponded to Supplementary Fig. 4B.

**Additional file 9: Table S1.** Peptide segments expressed by ARST theoretically and comparision in the swiss-prot database.

**Additional file 10: Table S2.** Proteins binding to ARST sense strands and anti-sense strands.

**Additional file 11: Table S3.** ALDOA truncation plasmid segments according to the catRAPID database.

**Additional file 12: Table S4.** Reagents and antibody information.

## Data Availability

The datasets during and/or analyzed during the current study are available from the corresponding author on reasonable request.

## Abbreviations

ARST: ALDOA-related specific transcript NONSHAT138818.2
ALDOA: Aldolase A
GBM: Glioblastoma multiforme
LGG: Low-grade glioma
FBS: Fetal bovine serum
DMEM: Dulbecco’s modified eagle medium
qRT-PCR: Quantitative real-time PCR
EdU: 5-ethynyl-2′-deoxyuridine
DAPI: 4′,6-diami-dino-2-phenylindole
BCA: Bicinchoninic acid
co-IP: Co-immunoprecipitation
IF: Immunofluorescence
NHA: Normal human astrocytes
ADP: Actin depolymerizing protein
AA: Amino acid
CFL: Cofilin
