# Supplementary figures and images for: A novel lncRNA ARST represses glioma progression by inhibiting ALDOA-mediated actin cytoskeleton integrity
Source: J Exp Clin Cancer Res. 2021 Jun 7;40:187. doi: 10.1186/s13046-021-01977-9 (PMC8183030; doi:10.1186/s13046-021-01977-9)

Supplementary Figure 1

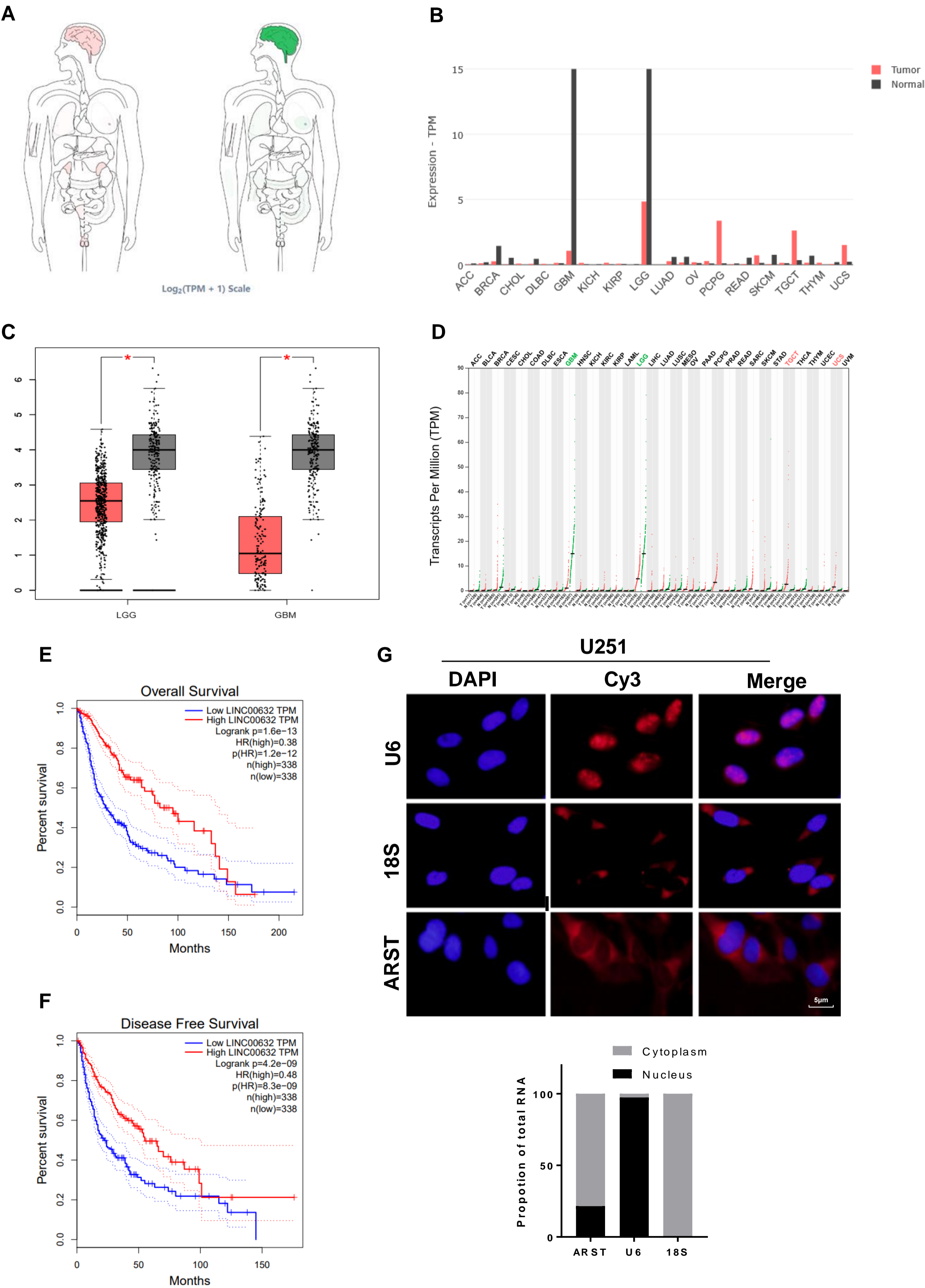

Supplement: Supplementary file 1 — Additional file 1: Figure S1. (A) The expressions of LINC00632 in normal people (left) and glioma patients (right) based on the TCGA database. (B) The differential expression levels of LINC00632 are displayed in different tumors compared with the respective normal tissues. (C) Graphic representation of relative LINC00632 expression level (TPM) in different tissues (GBM vs. GTEx, LGG vs. GTEx). GBM and LGG represented glioblastoma multiforme and low grade gliomas in the TCGA datasets. GTEx represented normal brain tissue in the GTEx database. (D) Transcripts per million (TPM) of LINC00632 in different cancers according to the TCGA database. GBM and LGG are highlighted in green (downregulated). (E) Overall and (F) disease free survivals of the glioma patients with relative low or high level of LINC00632 expressions were assessed in the GEPIA database (cut-off value is 50%). (G) Fluorescence in situ hybridization (FISH) assay was performed to detect the location of ARST in the U251 cells. Human 18S was used as a cytoplasm internal control and human U6 was used as a nucleus internal control. Proportions of ARST and the internal controls were determined in the cytoplasm and nucleus of the cells. Scale bar = 5 μm. [file 13046_2021_1977_MOESM1_ESM.pdf]

# Supplementary Figure 2

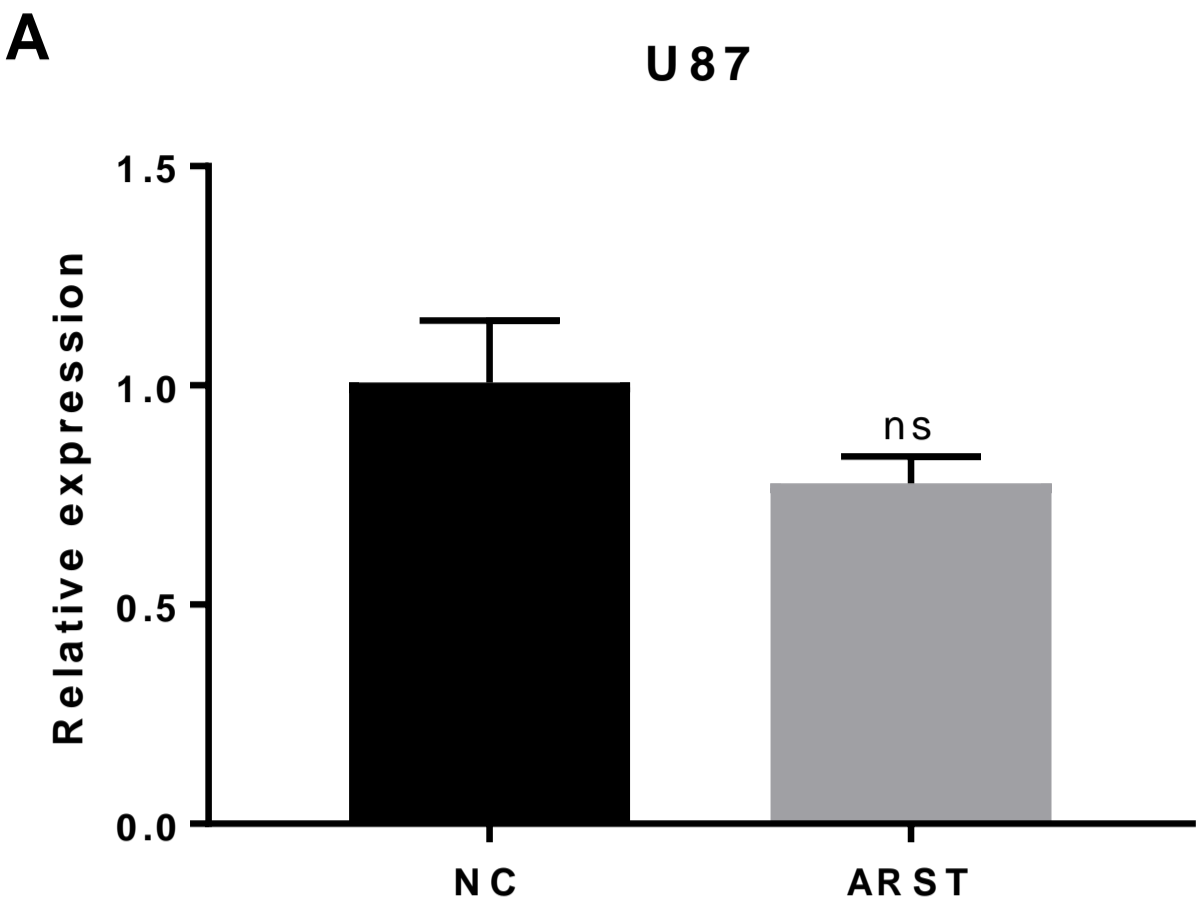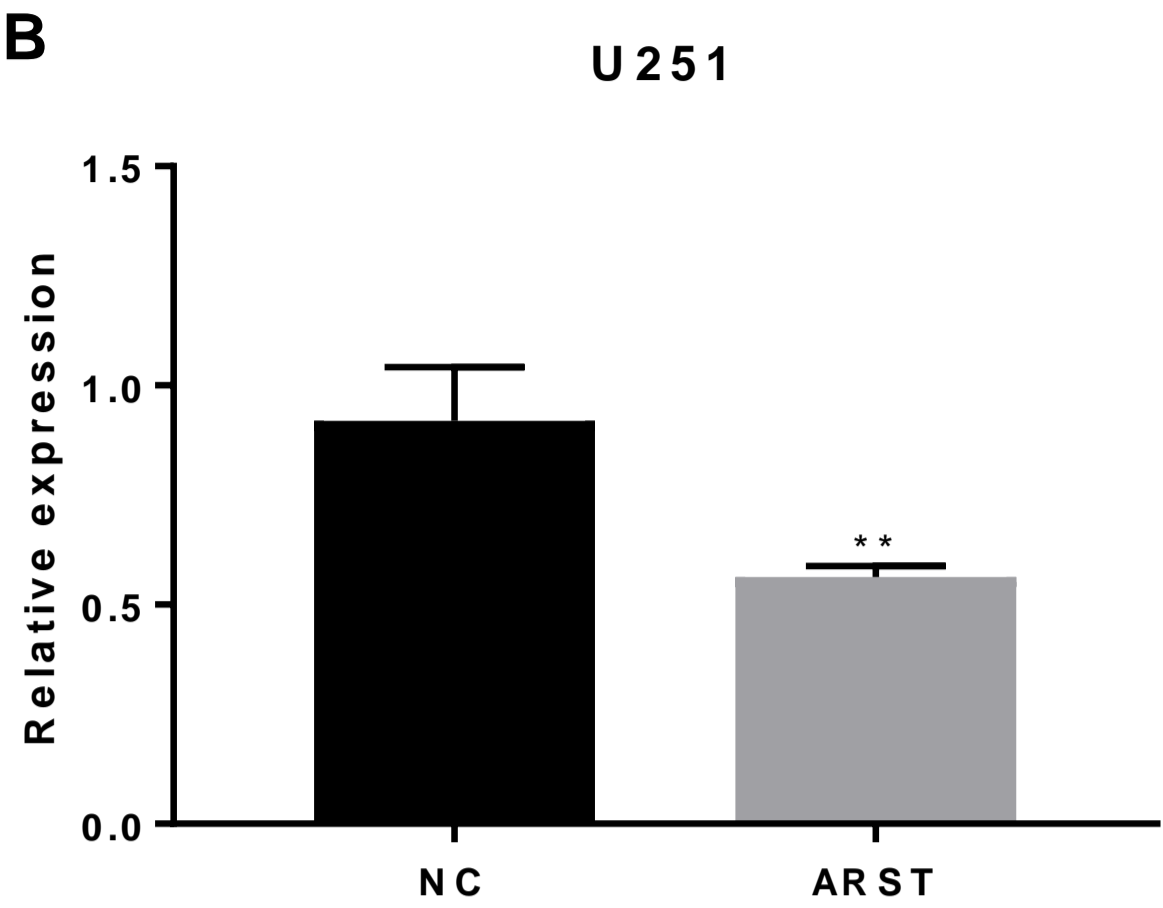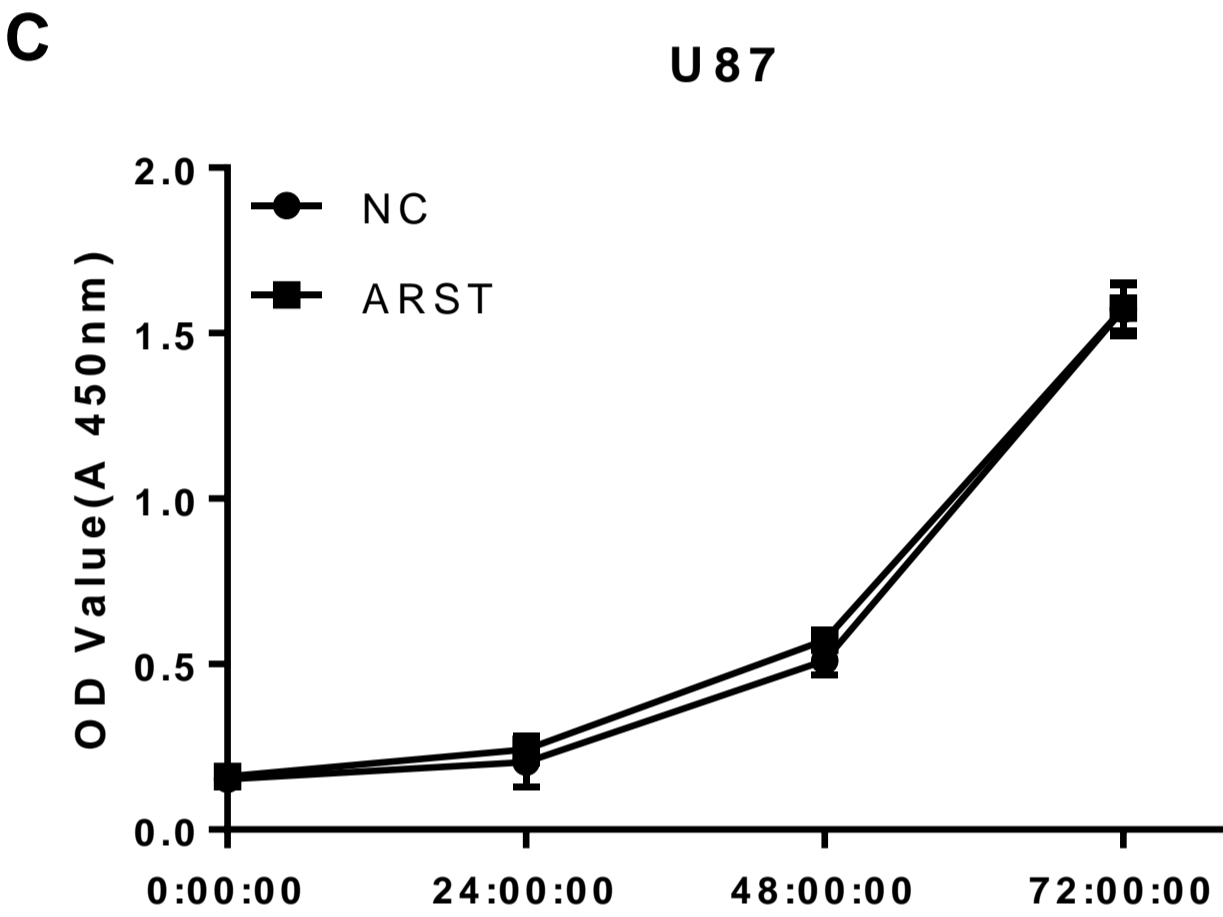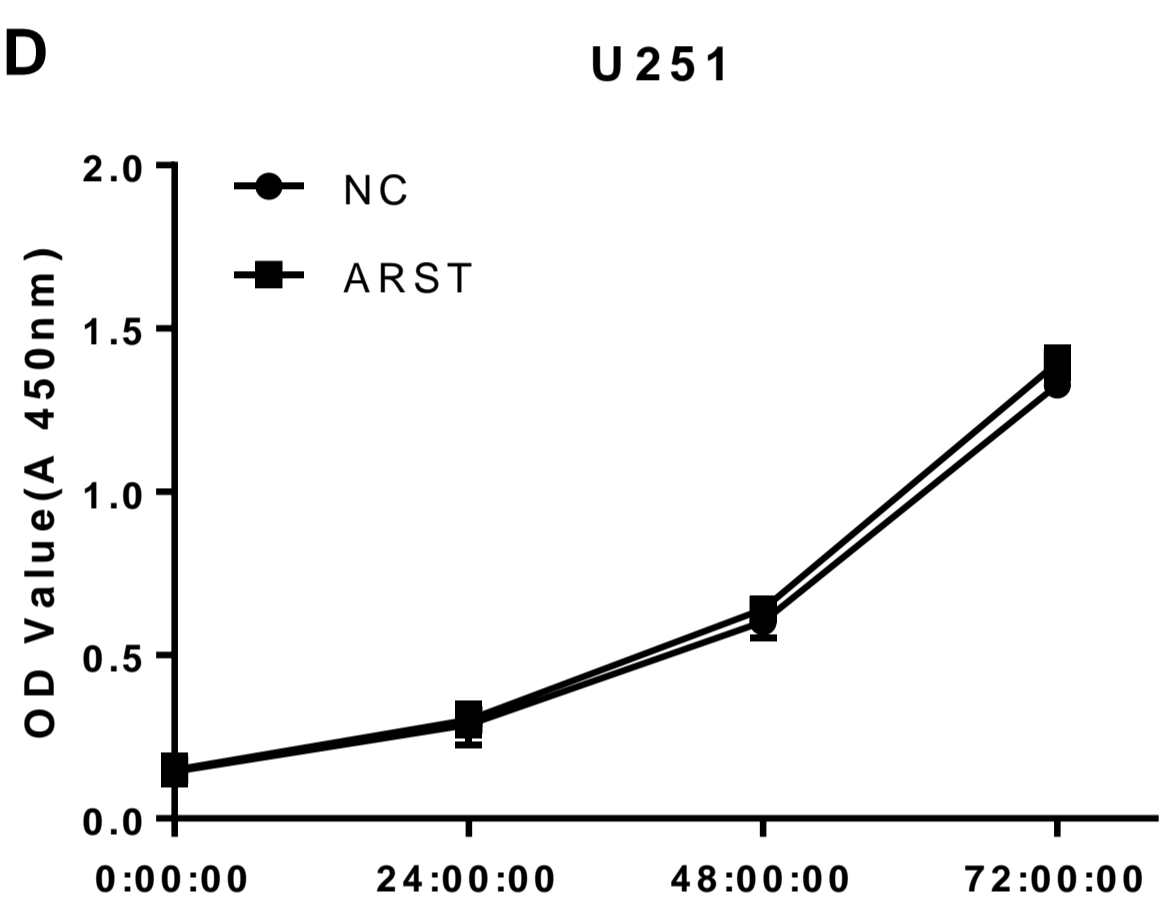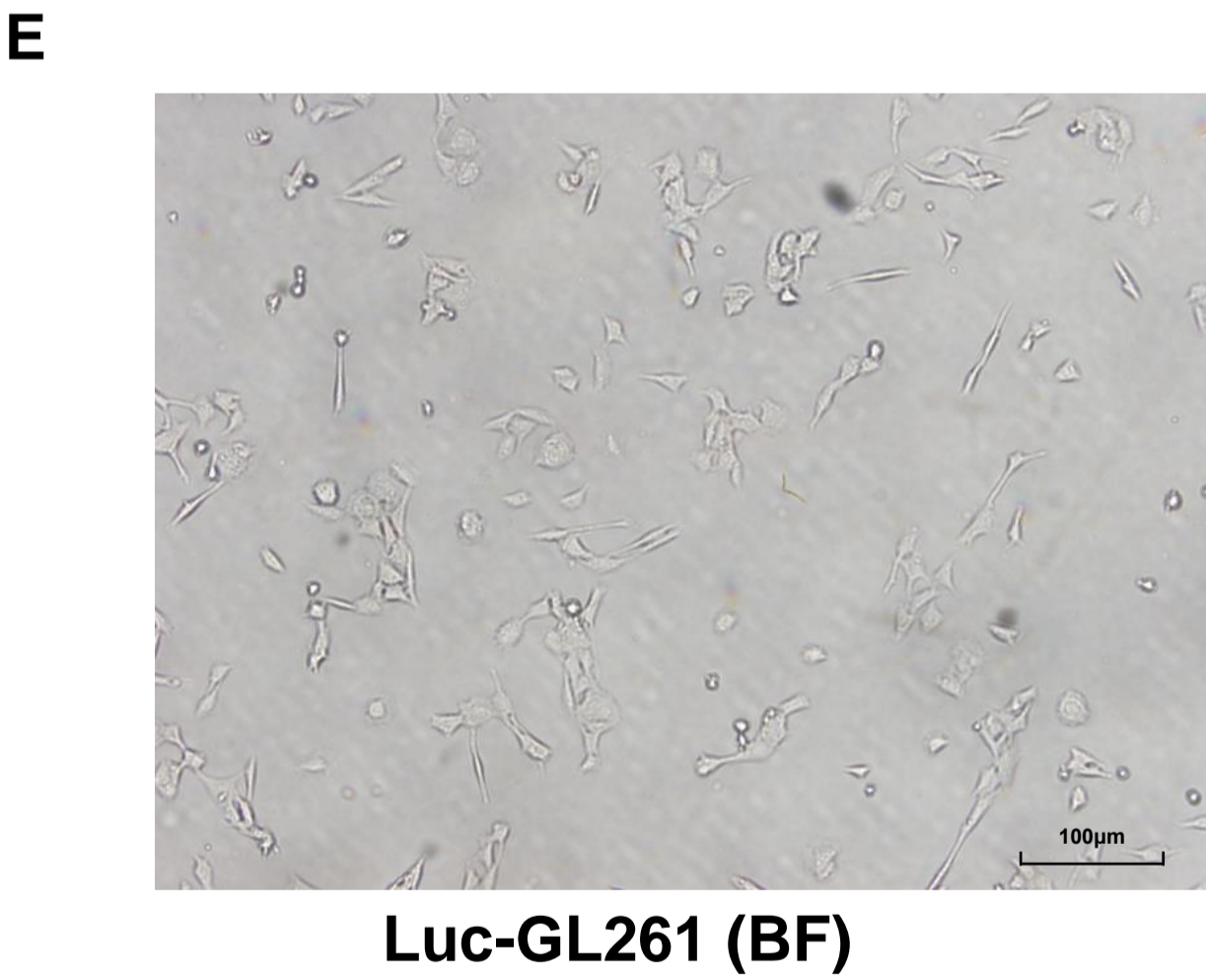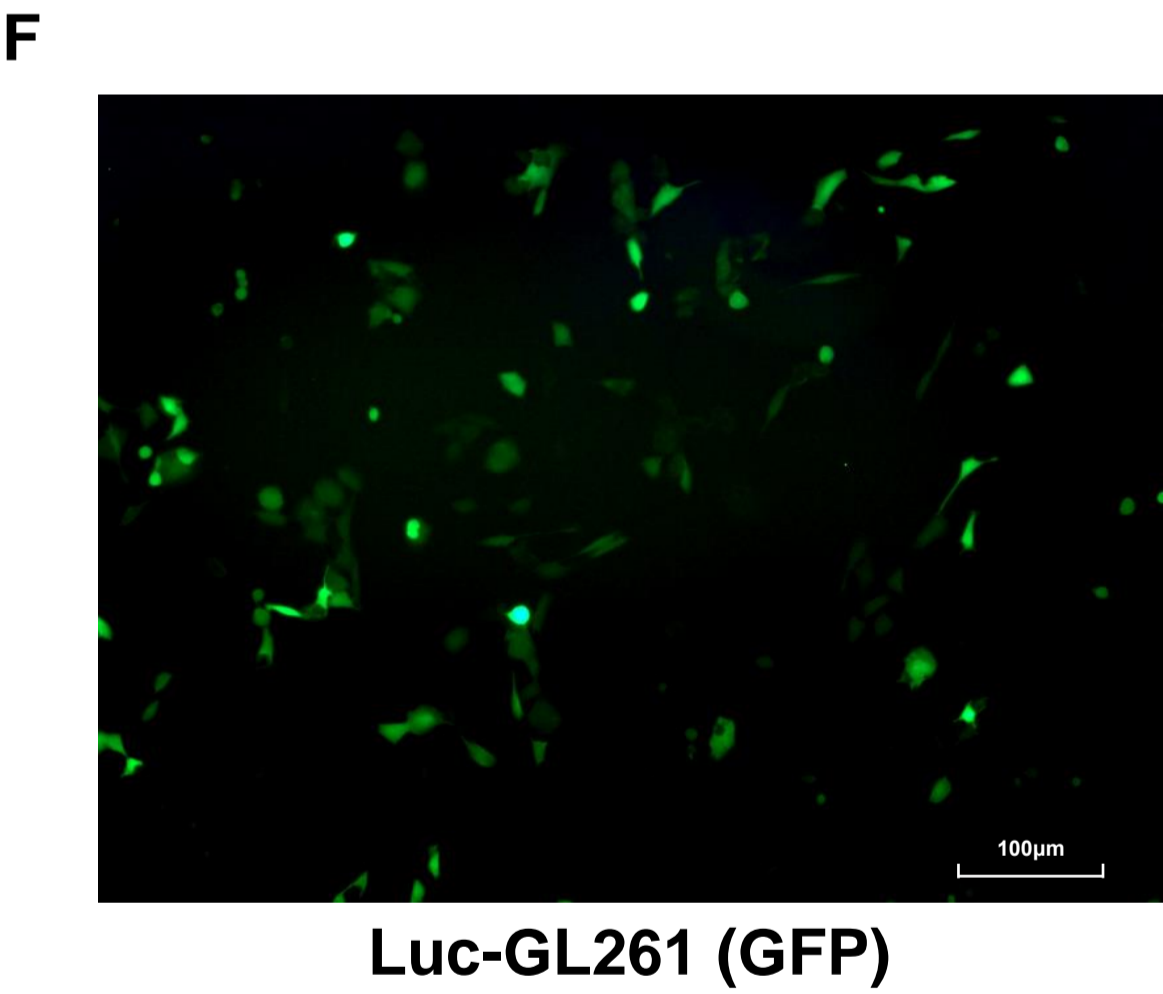

Supplement: Supplementary file 2 — Additional file 2: Figure S2. (A) The efficiencies of ARST knockdown in the U87MG and U251 cells were tested by qRT-PCR. **P < 0.01. (B) The growth curves of the transfected glioma cells were determined using CCK-8 assay. (C) Lentivirus infected GL261 cells together with GFP and luciferase were examined under immunofluorescent microscope. Scale bar = 100 μm. All results were represented mean ± s.d. from three independent experiments. [file 13046_2021_1977_MOESM2_ESM.pdf]

### Supplementary Figure 3

**A**      Input      ARST      Anti

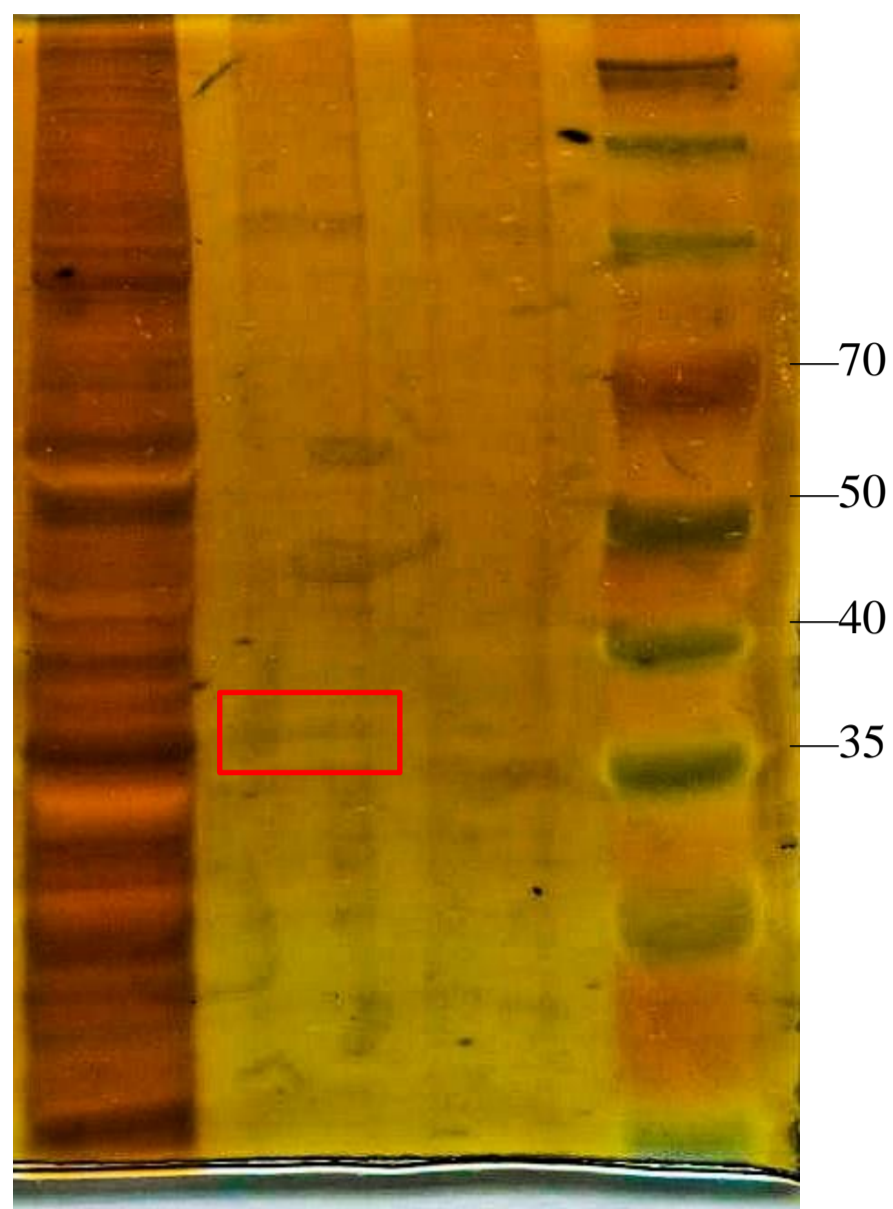

# B

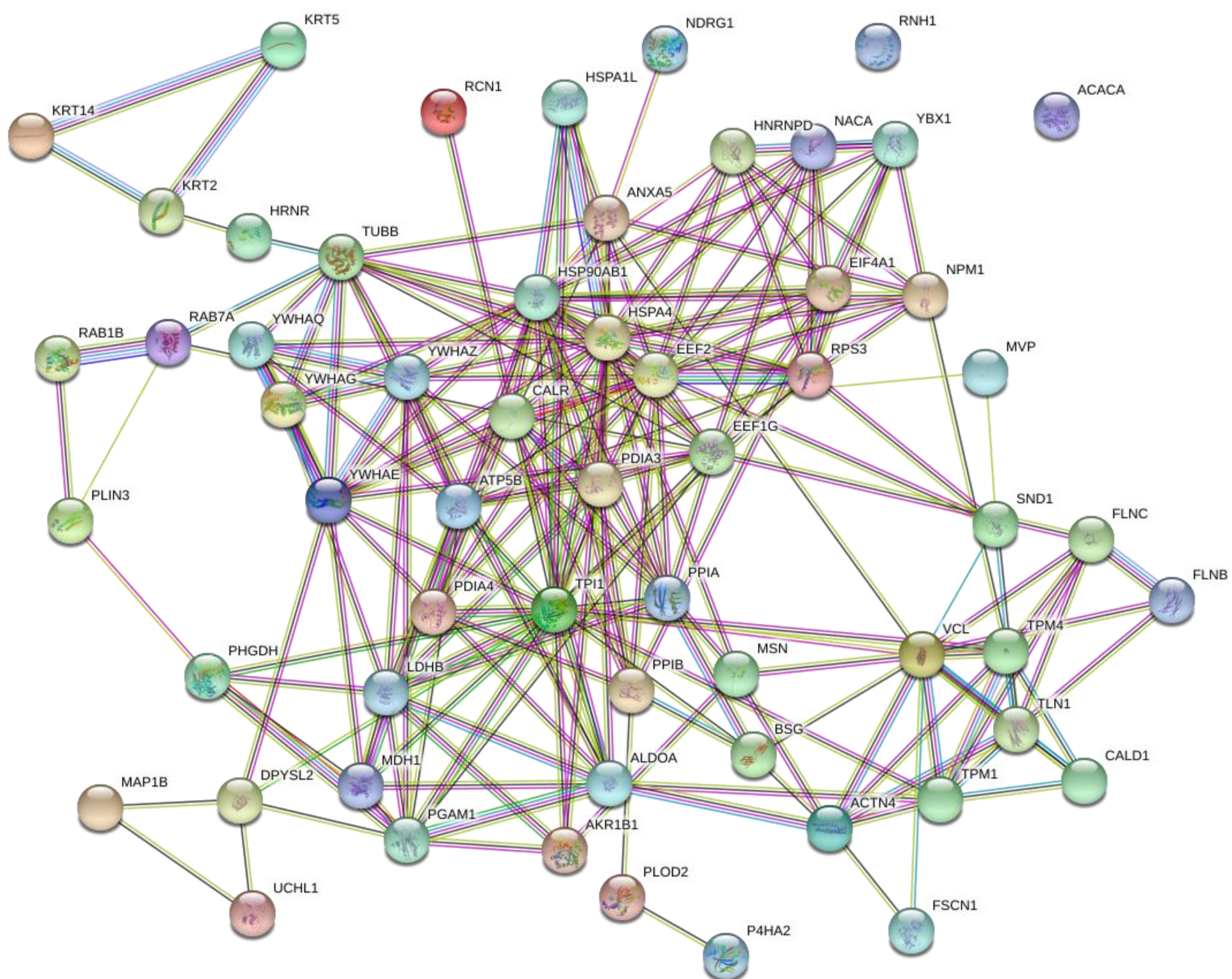

Supplement: Supplementary file 3 — Additional file 3: Figure S3. (A) Silver staining assay was performed to detect the eluted proteins following RNA pulldown assay using biotinylated sense and antisense strands of ARST. (B) The proteins that only bound to the sense strand of ARST were used to construct a PPI (Protein-protein interaction) network using the STRING database. [file 13046_2021_1977_MOESM3_ESM.pdf]

# Supplementary Figure 4

A

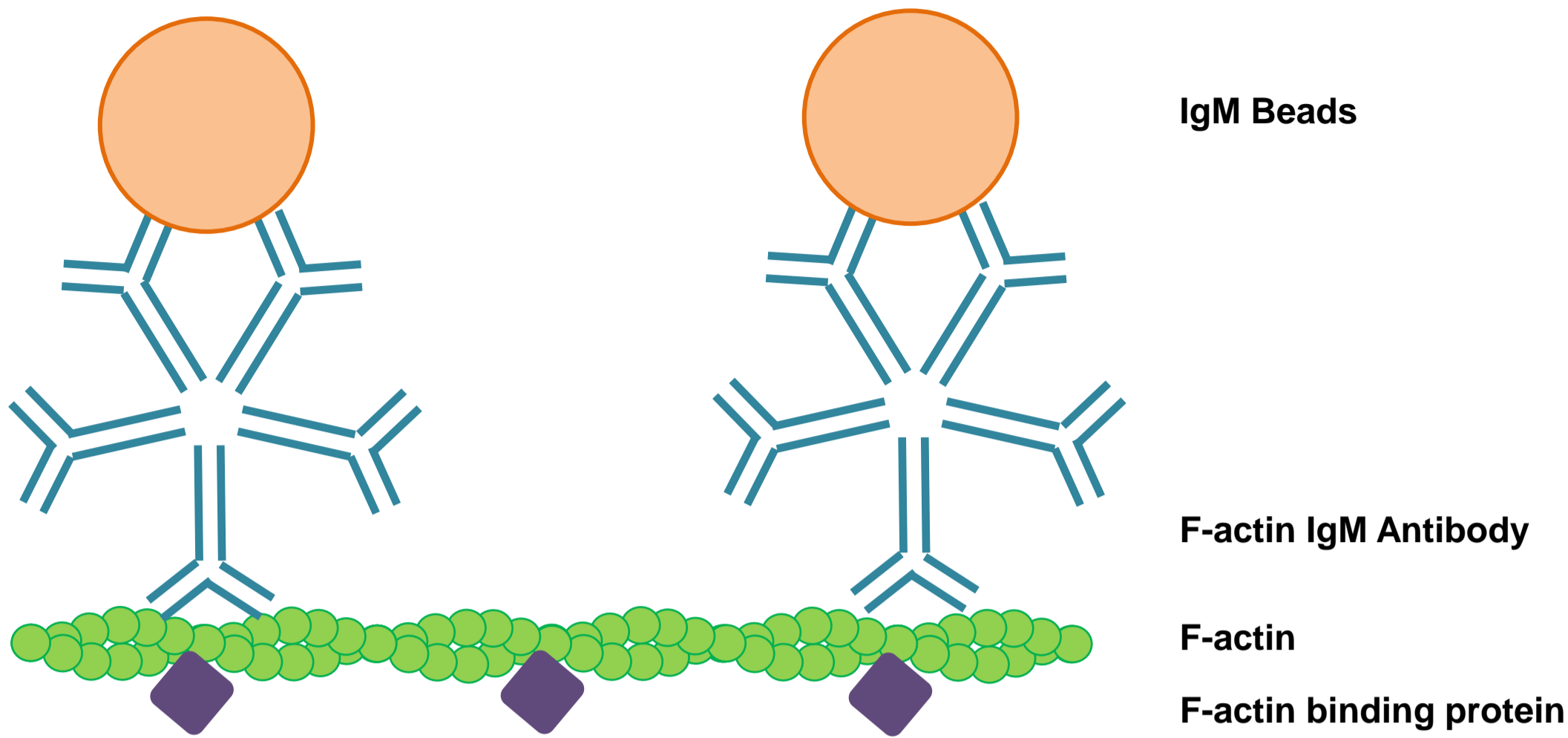

B

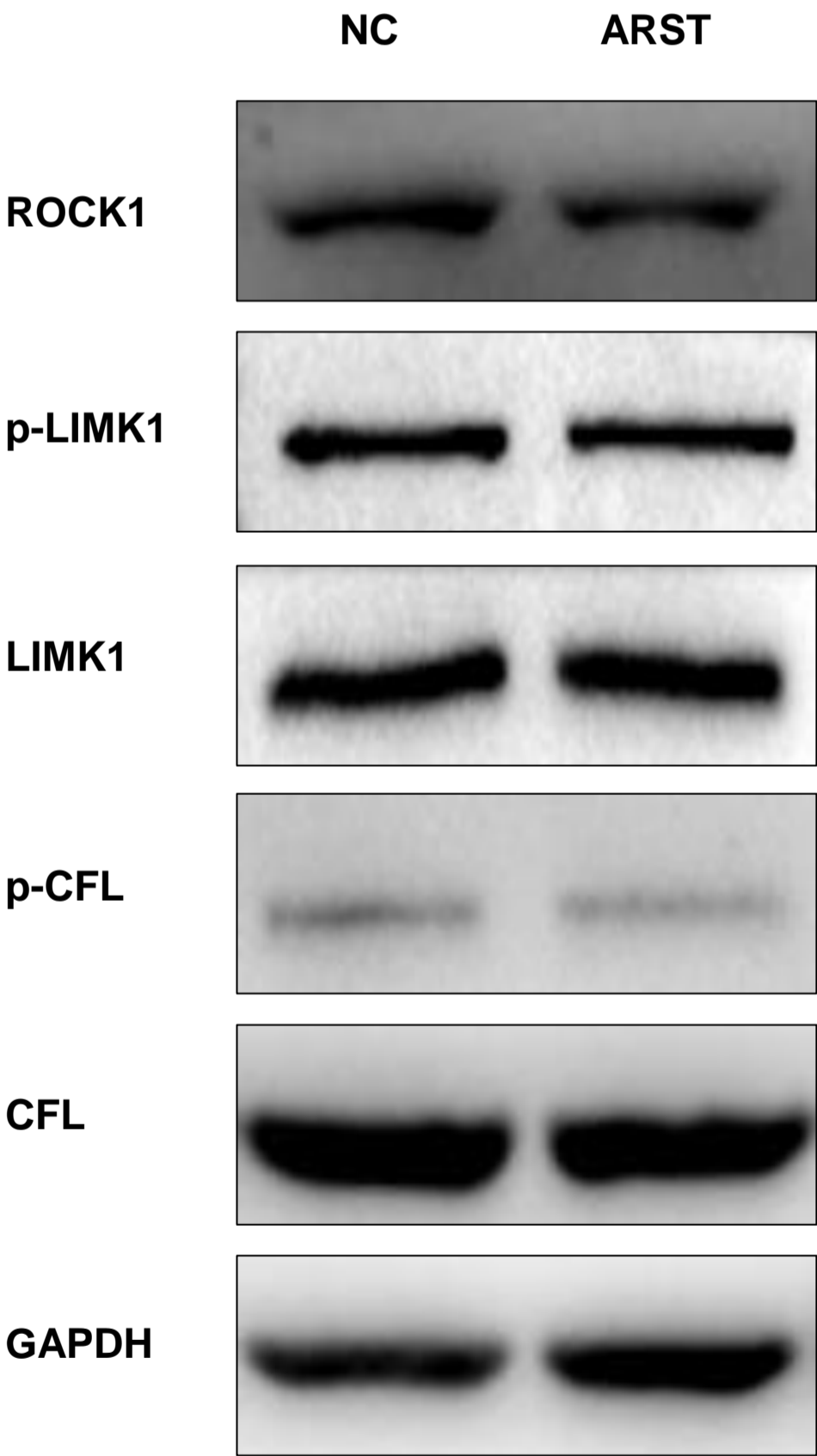

C

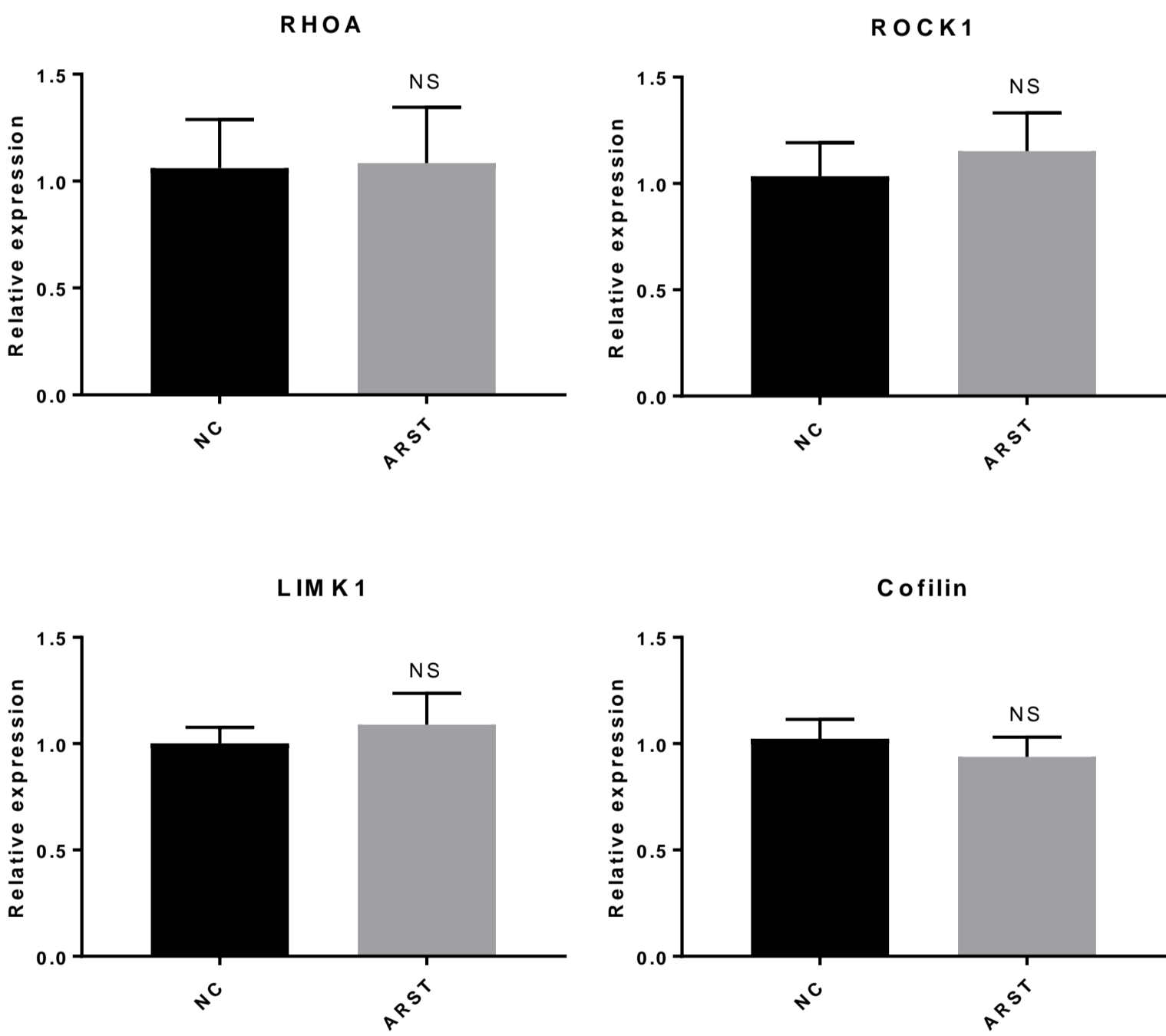

Supplement: Supplementary file 4 — Additional file 4: Figure S4. (A) The schematic diagram of F-actin immunoprecipitation. (B) Western blot analysis showed the changes of phosphorylation status of cofilin and LIMK1 in the U87MG cells after upregulation of ARST. GAPDH was used as the internal control. (C) qRT-PCR analysis was performed to show the mRNA levels of RhoA/ROCK1/LIMK/cofilin in the U87MG cells when ARST was overexpressed. [file 13046_2021_1977_MOESM4_ESM.pdf]

# Supplementary Figure 6

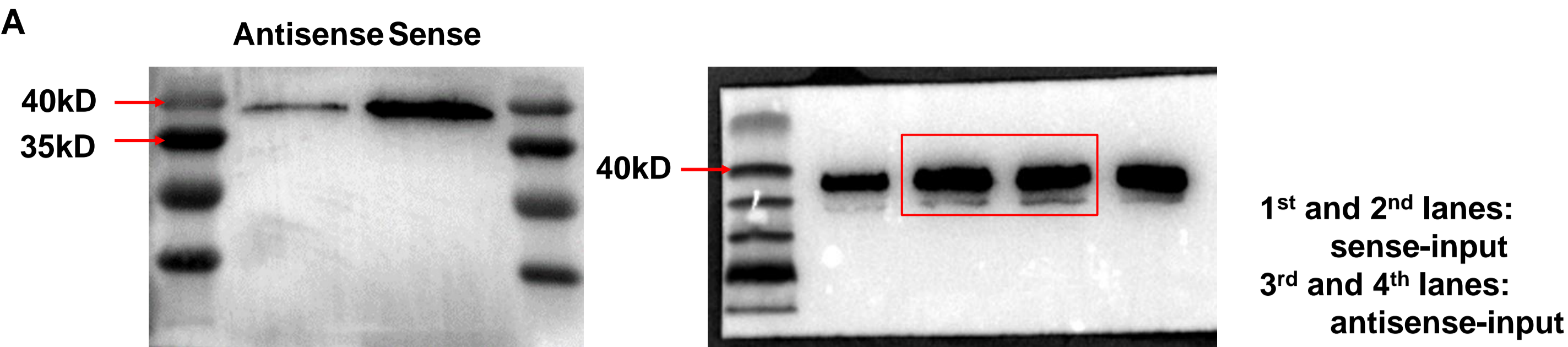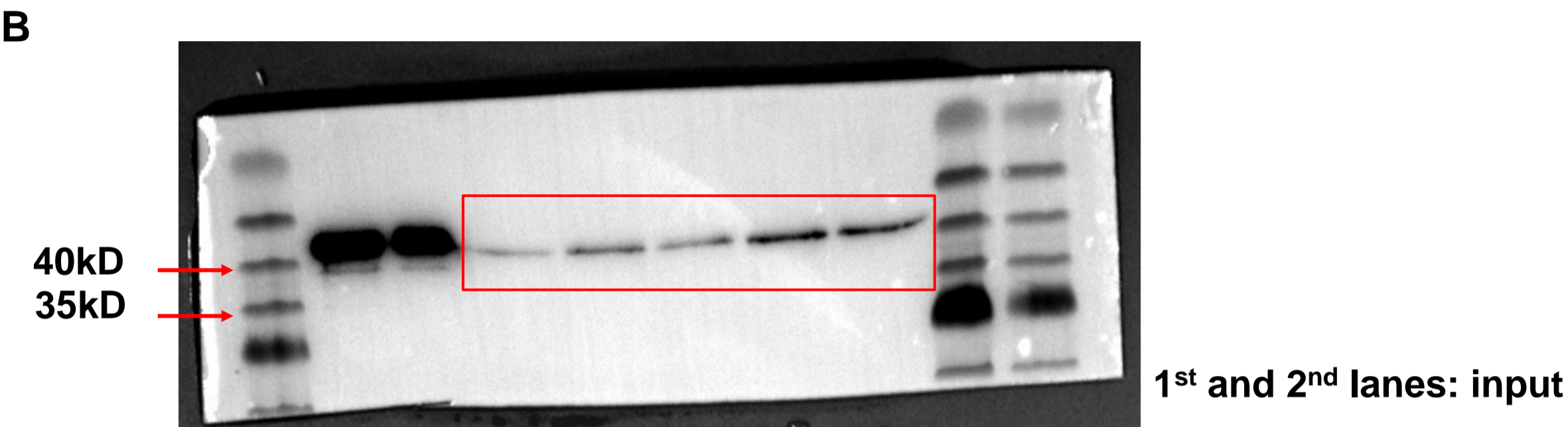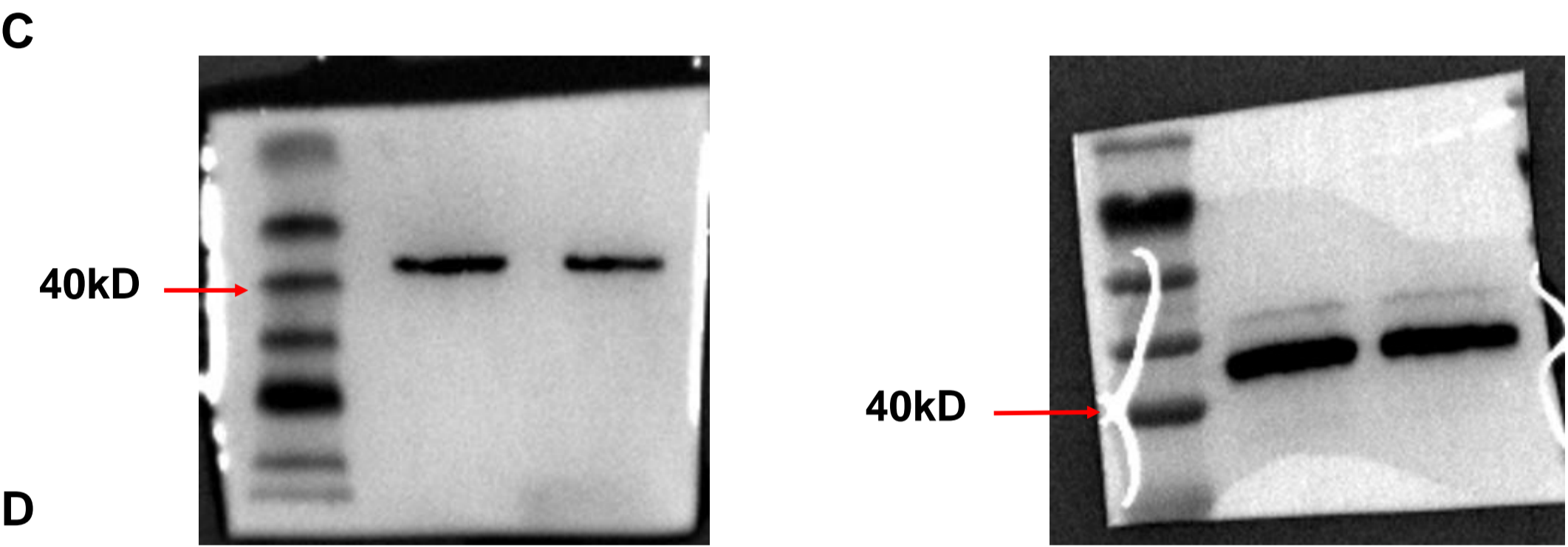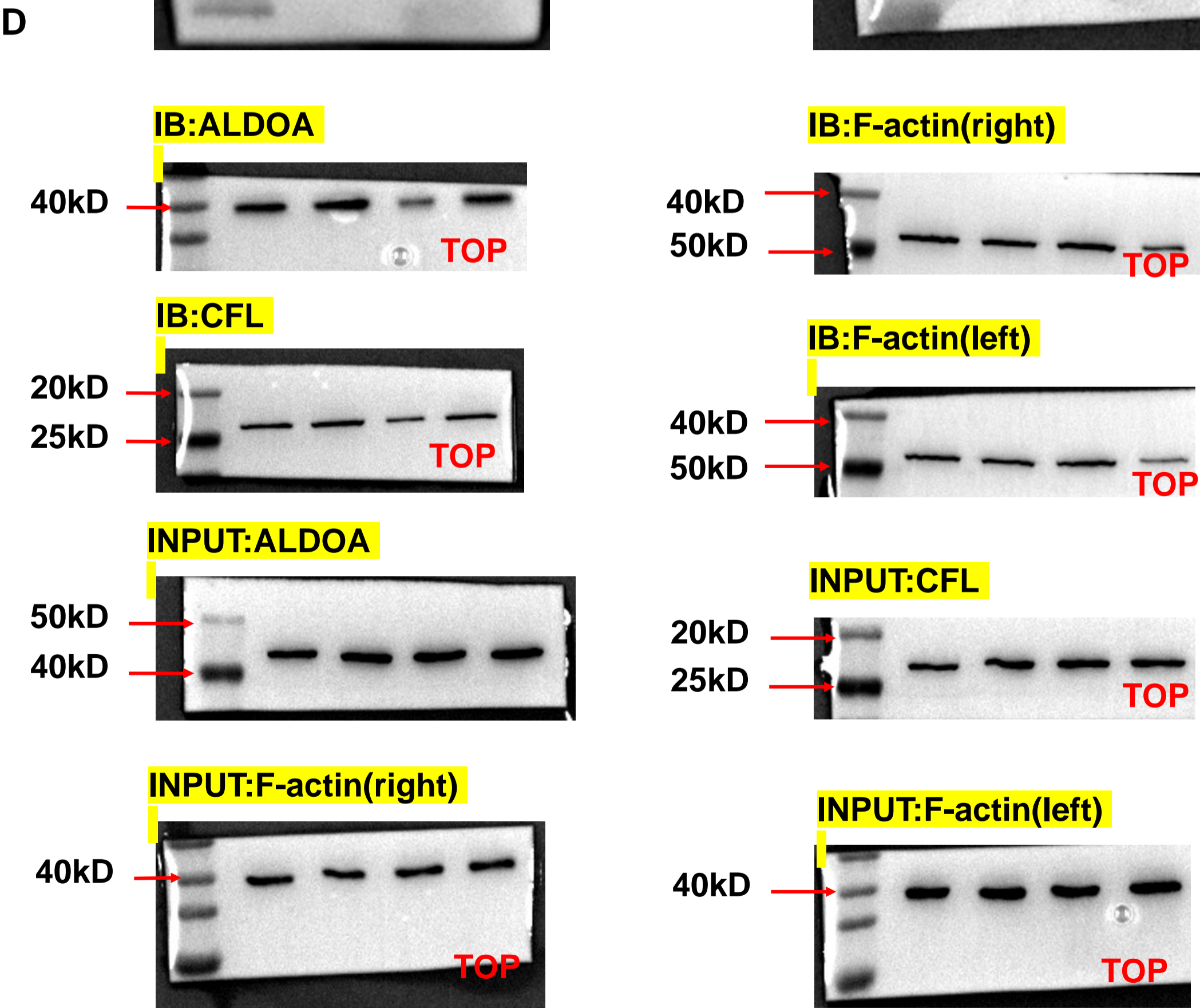

Supplement: Supplementary file 6 — Additional file 6: Figure S6 The original blot images in the manuscript, which corresponded to Fig. 3C (A), Fig. 3F (B),Fig. 3G (C) and Fig. 5A and B (D). [file 13046_2021_1977_MOESM6_ESM.pdf]

# Supplementary Figure 7

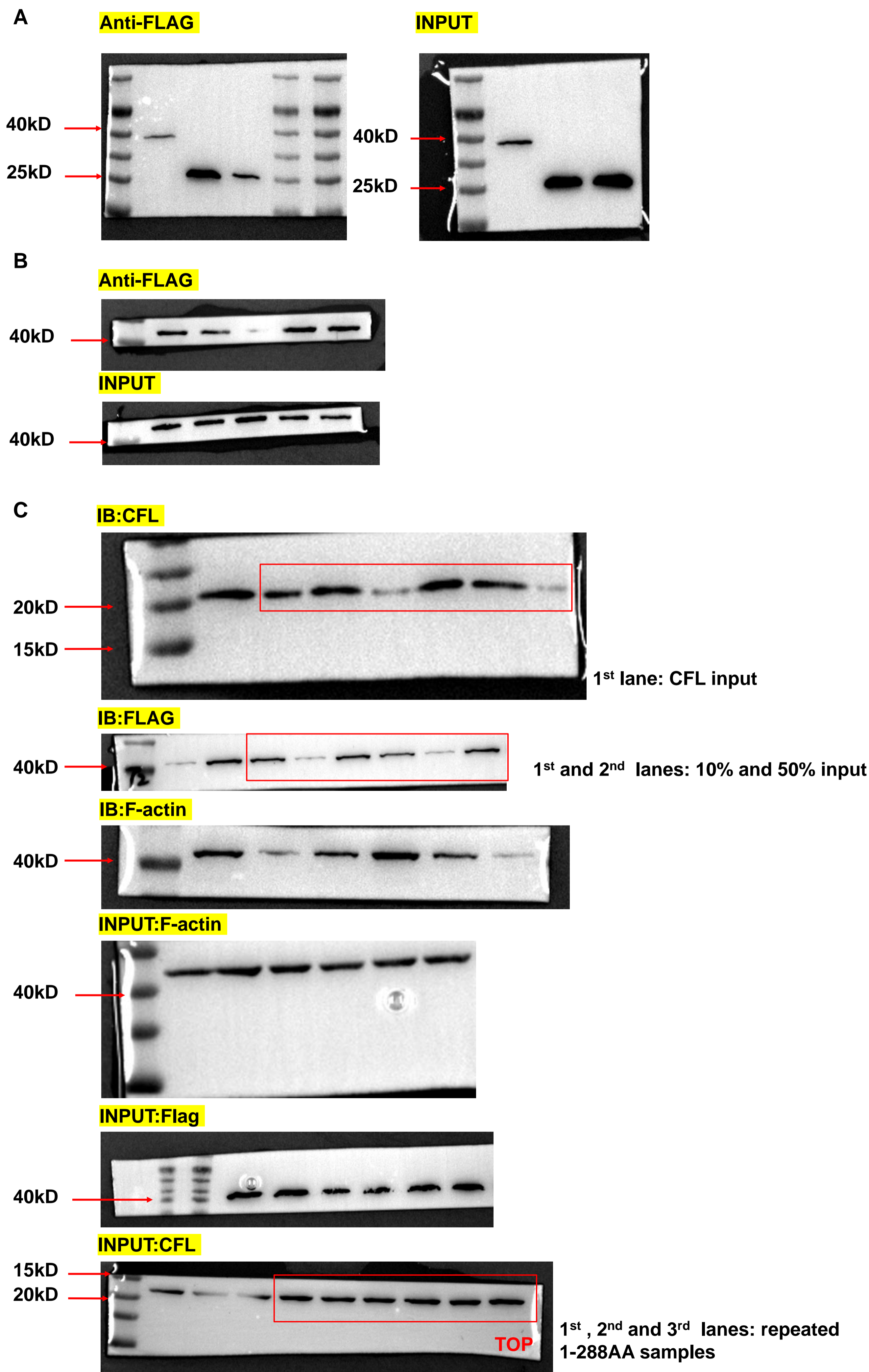

Supplement: Supplementary file 7 — Additional file 7: Figure S7. The original blot images in the manuscript, which corresponded to Fig. 6D (A), Fig. 6F (B) and Fig. 6G (C). [file 13046_2021_1977_MOESM7_ESM.pdf]
