# Supplementary material for: A novel lncRNA ARST represses glioma progression by inhibiting ALDOA-mediated actin cytoskeleton integrity
Source: J Exp Clin Cancer Res. 2021 Jun 7;40:187. doi: 10.1186/s13046-021-01977-9 (PMC8183030; doi:10.1186/s13046-021-01977-9)

Supplementary Figure 5

A

#	Protein region	RNA region	Interaction Propensity	Discriminative Power	Normalized Score
1	289-340	1276-1361	6.08	22	2.78
2	289-340	337-422	5.08	22	2.59
3	289-340	715-800	3.33	20	2.26
4	26-77	337-422	2.95	17	2.19
5	226-277	337-422	2.95	17	2.19
6	251-302	337-422	2.79	17	2.16
7	289-340	631-716	2.44	17	2.09
8	289-340	169-254	2.25	17	2.06
9	189-240	337-422	2.14	17	2.04
10	289-340	1948-2033	2.07	17	2.02
11	226-277	631-716	2.07	17	2.02
12	176-227	631-716	2.05	17	2.02
13	26-77	1276-1361	1.96	17	2.00
14	189-240	631-716	1.96	17	2.00
15	26-77	631-716	1.88	17	1.99
16	251-302	631-716	1.84	17	1.98
17	289-340	1696-1781	1.81	17	1.98
18	289-340	1681-1766	1.80	17	1.97
19	289-340	1360-1445	1.76	17	1.97
20	126-177	631-716	1.74	17	1.96

B

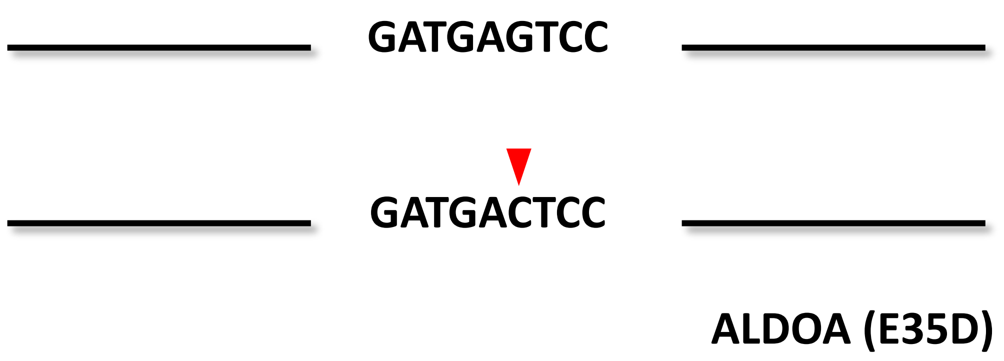

C

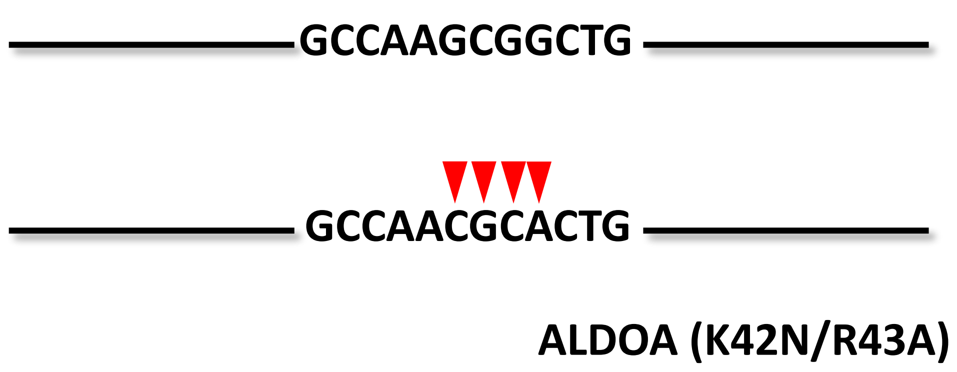

D

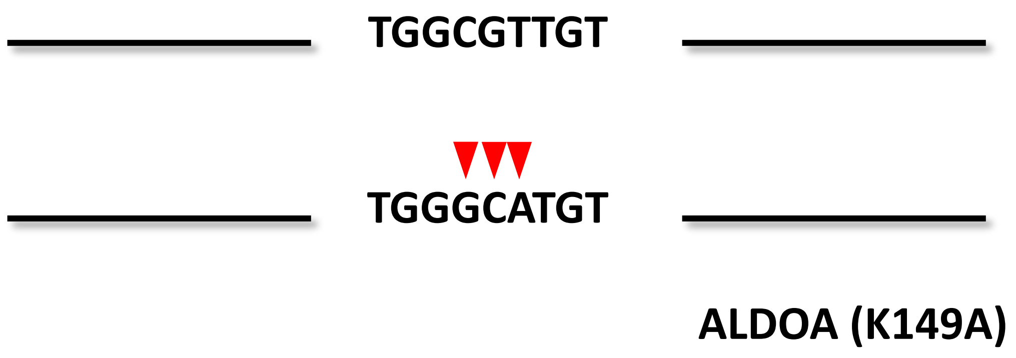

E

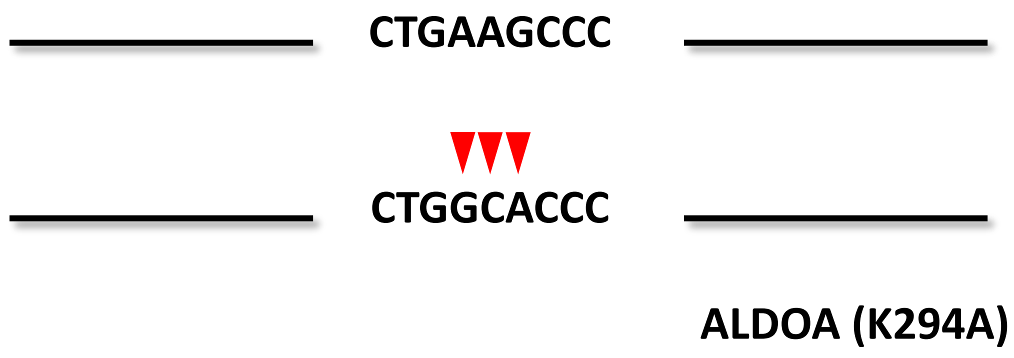

Supplement: Supplementary file 5 — Additional file 5: Figure S5. (A) The catRAPID online database was utilized to predict the binding domains of ALDOA and ARST. Interaction propensities of different regions were shown. (B-E) the specific sites of ALDOA that were mutated in the study were demonstrated. [file 13046_2021_1977_MOESM5_ESM.pdf]
