# Supplementary material for: A novel lncRNA ARST represses glioma progression by inhibiting ALDOA-mediated actin cytoskeleton integrity
Source: J Exp Clin Cancer Res. 2021 Jun 7;40:187. doi: 10.1186/s13046-021-01977-9 (PMC8183030; doi:10.1186/s13046-021-01977-9)
Supplement: Supplementary file 8 — Additional file 8: Figure S8 The original blot images in the manuscript, which corresponded to Supplementary Fig. 4B. [file 13046_2021_1977_MOESM8_ESM.pdf]

Supplementary Figure 8

A

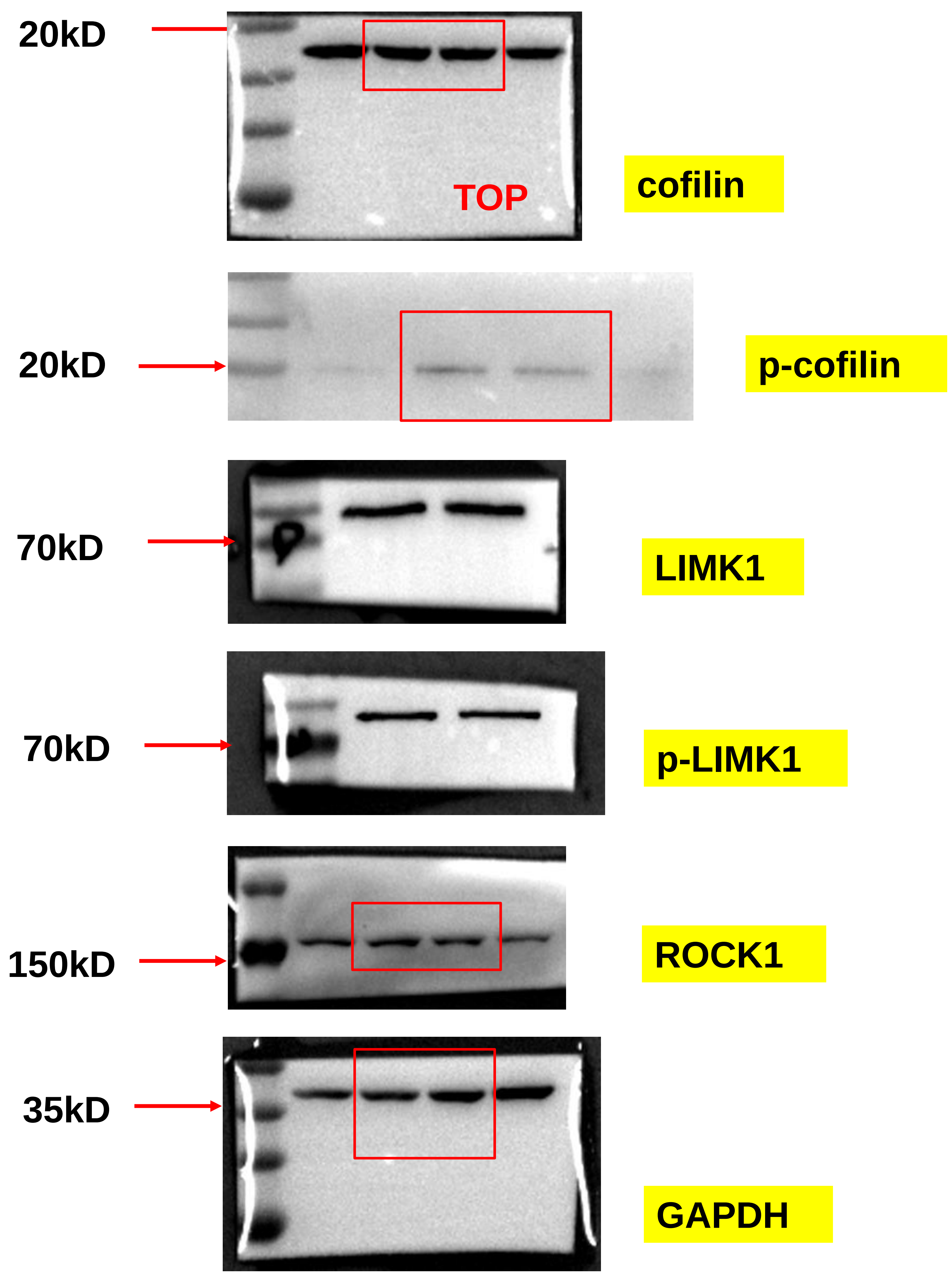

For four lanes membrane: 1st and 2nd lanes-repeated control cell lysate
3rd and 4th lanes- repeated overexpression cell lysate
